# Stabilizing Frustrated Phase Transitions in Selective Oxidation Reactions

**DOI:** 10.1002/adma.202515292

**Published:** 2025-11-24

**Authors:** Luis Sandoval‐Diaz, Thomas Götsch, Daniel Cruz, Maurits Vuijk, Juan M. Lombardi, Markus Pietsch, Kassiogé Dembélé, Adnan Hammud, Karsten Reuter, Christoph Scheurer, Axel Knop‐Gericke, Thomas Lunkenbein

**Affiliations:** ^1^ Fritz‐Haber‐Institute of the Max‐Planck‐Society 14195 Berlin Germany; ^2^ Department of Chemistry and Catalysis Research Center, TUM School of Natural Sciences Technical University of Munich 85748 Garching Germany; ^3^ Department of Heterogeneous Reactions Max Planck Institute for Chemical Energy Conversion 45470 Mülheim an der Ruhr Germany; ^4^ Bavarian Center for Battery Technology (BayBatt) University of Bayreuth 95448 Bayreuth Germany; ^5^ Chair of Operando Analytics for Electrochemical Energy Storage University of Bayreuth 95448 Bayreuth Germany

**Keywords:** frustrated phase transition, operando spectromicroscopy, selectivity control, selective oxidation

## Abstract

Frustrated phase transitions represent the ideal working state of a heterogeneous catalyst. These states exist within a narrow parameter window, making them difficult to stabilize. Here, it is shown for the selective oxidation of 2‐propanol to acetone over Co_3_O_4_ spinels that the addition of water extends the stability regime of the relevant frustrated phase transition. This conclusion is based on results obtained from multi‐modal experiments, including *operando* scanning electron microscopy (OSEM), near ambient pressure X‐ray photoelectron spectroscopy (NAP‐XPS), transmission electron microscopy (TEM), and computer vision analysis. It is found that the most selective state for acetone formation coincides with a dynamic spinel structure that fluctuates through reversible redox processes. At elevated temperatures, this metastable state undergoes a complete phase transition into the rock‐salt CoO phase characterized by low acetone selectivity. This process is found to be mediated by the generation of mobile vacancies. The addition of water vapor mitigates vacancy mobility and stabilizes the selective, but thermodynamically frustrated, state. As such, the study conceptualizes a strategy to extend the lifetime of a catalyst during reaction by the adequate addition of a co‐reactant.

## Graphical abstract

Using multimodal operando spectromicroscopy, the frustrated phase transition responsible for selective oxidation over cobalt oxide spinel catalysts is identified, corresponding to combined surface reaction and Mars‐van Krevelen (MvK) dynamics. This regime is disrupted by the formation of mobile vacancies and the depletion of active oxygen at high temperatures. Co‐feeding water vapor mitigates these effects, thereby enhancing both selectivity and lifetime.

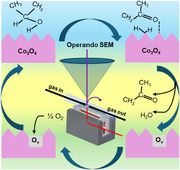

## 1 Introduction

Catalytic reactions rely on the cyclical renewal of active sites, moderated by the local chemical potential.^[^ ^1^ ^]^ From a macroscopic perspective, this renewal may happen very rapidly and through multiple paths, giving the impression that the active sites simultaneously exist in several configurations statistically distributed in space and time.^[^ ^2^, ^3^ ^]^ Microscopically, the active sites are dynamic and continuously fluctuate within excited regimes without fully transitioning into any thermodynamically stable state. This dynamic behavior can be described as a frustrated phase transition,^[^ ^1^, ^4^, ^5^ ^]^ where competing interactions that cannot be simultaneously satisfied prevent the system from relaxing into a single, stable, yet catalytically inactive, state. In redox processes, such frustration is evinced by coexisting oxidized and reduced phases that are unable to reorganize into the most stable configuration.^[^ ^6^ ^]^ Hence, the sites undergo a sustained alternation in the oxidation states during the catalytic cycle, which can lead to long‐range phenomena such as surface patterns,^[^ ^7^ ^]^ chemical waves ^[^ ^8^, ^9^ ^]^ and self‐sustained oscillations.^[^ ^3^ ^]^

During a reaction, the highest catalytic performance often occurs when the catalyst approaches, but does not complete, a reaction‐induced phase transition.^[^ ^2^ ^]^ In heterogenous redox reactions, this may correspond to a surface that is nearly fully oxidized or fully reduced by the chemical potential, although still remains within the frustrated regime. If this frustrated regime is surpassed and the system completes the phase transition, the catalytic performance usually drops. Therefore, maintaining the catalyst in this dynamic regime while actively impeding the completion of the phase transformation could be a strategy to optimize the overall catalytic performance.

In this context, the selective oxidation of small organic molecules can give important insights into the role of these frustrated transitions in heterogeneous catalysis.^[^ ^10^, ^11^ ^]^ In selective oxidation, organic molecules are converted into partially oxidized intermediates without fully oxidizing them.^[^ ^10^ ^]^ The process aims to convert a stable molecule into a valuable, but less stable, product, useful in fine chemicals, pharmaceuticals, or energy storage applications. The success of these transformations largely depends on the kinetic control and, therefore, on a delicate balance between the chemical potential and the properties of the catalyst.^[^ ^10^, ^12^ ^]^ Noble metals such as Au, Pt, and Pd,^[^ ^13^, ^14^, ^15^, ^16^ ^]^ are commonly employed in selective oxidation due to their high activity. Additionally, oxides of several transition metals ^[^ ^17^ ^]^ including vanadium and mixed vanadium‐molybdenum,^[^ ^16^, ^17^, ^18^, ^19^, ^20^ ^]^ mixed manganese‐anatase,^[^ ^21^ ^]^ nickel oxide,^[^ ^22^ ^]^ and several perovskite materials have also been used.^[^ ^23^, ^24^ ^]^ Among these, cobalt oxide spinel (Co_3_O_4_) catalysts have demonstrated promising properties thanks to their high activity and stability, resistance to poisons, and low cost.^[^ ^11^, ^25^, ^26^ ^]^

It has been shown that cobalt oxide spinel catalysts are very active for the gas‐phase oxidation of 2‐propanol to acetone.^[^ ^9^, ^25^ ^]^ However, it remains unclear whether this activity originates from the spinel ability to dissociate oxygen, its role as a mobile oxide carrier, or by the presence of under‐stoichiometric surfaces with oxygen vacancies acting as binding sites.^[^ ^11^ ^]^ Furthermore, it has recently been observed by *operando* TEM imaging the coexistence of CoO nanoparticles and internal voids at the CO_3_O_4_ spinel during the selective oxidation of 2‐propanol.^[^ ^28^ ^]^ This simultaneous presence of several phases may involve a complex interaction of the material with the chemical potential, offering a unique opportunity to investigate the role of frustrated phase transitions in the reaction kinetics. Additionally, while the reaction has been characterized up to 300 °C, the selective oxidation behavior and associated catalytic dynamics at higher temperatures remain unexplored. Understanding the behavior of this system at elevated temperatures is relevant for devising strategies to improve the catalyst stability at increasingly higher conversion levels,^[^ ^11^, ^25^ ^]^ Furthermore, the reaction selectivity depends on the redox and acid‐base properties of the catalyst (**Table** **1**). Hence, it is crucial to decipher how the catalyst properties are modulated by the frustrated phase transitions inherent to the active regime, aiming at a rational control of the catalytic operating window and lifetime.

**Table 1:** A compilation of the characteristic catalytic reactions of 2‐propanol with and without O_2_.

Reactant	Products	Type of reaction	Catalyst
(CH_3_)_2_CHOH	(CH_3_)_2_C=O + H_2_	Dehydrogenation	Noble metals
(CH_3_)_2_CHOH + 1/2 O_2_	(CH_3_)_2_C=O + H_2_O	Oxidative dehydrogenation	Noble metals Transition metal oxides
(CH_3_)_2_CHOH	CH_3_‐CH=CH_2_ + H_2_O	Dehydration	Strong acids
(CH_3_)_2_CHOH + 9/2 O_2_	3CO_2_ + 4H_2_O	Total oxidation	Noble metals

In this sense, previous studies have suggested that co‐feeding water may enhance acetone selectivity due to the competitive adsorption on strong acidic sites, preventing the alcohol dehydration.^[^ ^29^ ^]^ However, research on multinary oxide catalysts has shown that co‐feeding water can be a bane or a boon for the performance, depending on the catalyst composition.^[^ ^24^, ^25^, ^26^ ^]^ This observation implies that the role of water in the reaction mechanism is not completely clear.

In this study, we investigate the evolution of Co_3_O_4_ catalysts during the gas‐phase oxidation of 2‐propanol using a combination of *operando* scanning electron microscopy (OSEM).^[^ ^3^, ^30^ ^]^ near‐ambient pressure X‐ray photoelectron spectroscopy (NAP‐XPS), transmission electron microscopy (TEM), computer vision investigations,^[^ ^31^ ^]^ and theoretical calculations. Our findings reveal the correlation between the catalyst structure and the product distribution as a function of the reaction temperature, up to 500 °C. We found that the acetone was selectively produced as long as the spinel bulk structure was preserved. However, at temperatures above 375 °C, the spinel catalyst transformed into a CoO‐enriched material with internal voids, corroborating previous investigations.^[^ ^28^, ^32^ ^]^ This phase transformation was accompanied by a marked decay of the acetone selectivity. Our results suggest that the selective regime is characterized by a combination of surface reaction and Mars‐van Krevelen (MvK) mechanisms of acetone formation.^[^ ^10^, ^11^, ^33^, ^34^ ^]^ During the MvK activation, vacancies are continuously generated and replenished. The depletion of the catalyst oxygen and the mobilization of these vacancies into the bulk were identified as key factors driving the phase transition from the spinel structure into the less selective rock‐salt (CoO_x_) phase. Co‐feeding of water vapor stabilized the selective frustrated regime by rapid refilling of the vacancies and an increased oxygen potential, impeding the phase transformation towards CoO_x_. Our results show that using water as a co‐feed extended the temperature range and enhanced the rate of selective acetone production in this system.

## 2 Results

### 2.1 Catalyst Preparation

**Figure 1:**
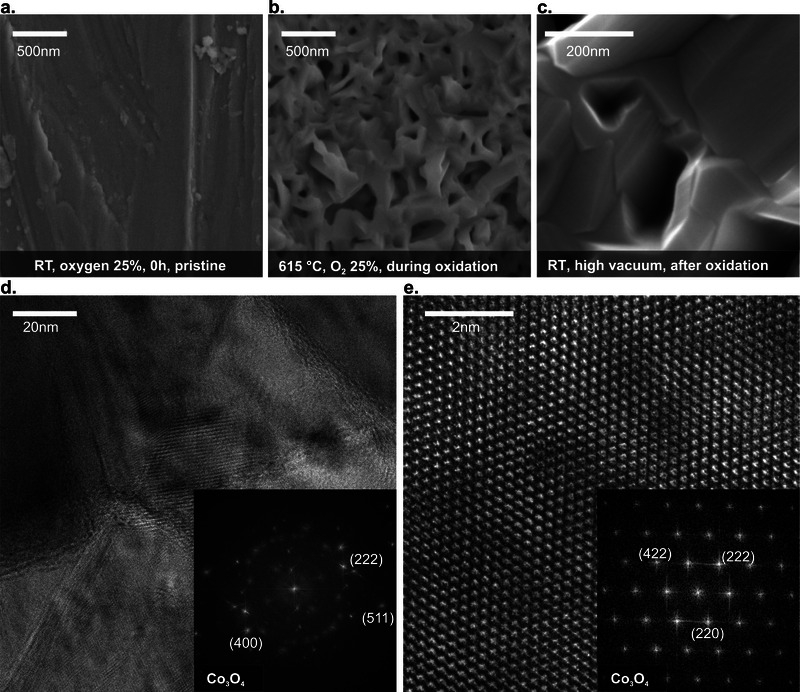
Electron microscopy images illustrating the preparation and the structure of the cobalt oxide catalyst. a) An in situ SEM image of the starting metallic cobalt foil (71.2 mg) at room temperature in a flow of Ar 3mL_N_min^−1^: O_2_ 1mL_N_min^−1^ and a chamber pressure of 32 Pa. b) An in situ SEM image of the catalyst surface during treatment at 615 °C in a flow of Ar 3mL_N_min^−1^: O_2_ 1mL_N_min^−1^ and a chamber pressure of 32 Pa. c) An SEM image of the pristine oxide surface revealing the intricate internal microstructure. d) A TEM (bright‐field) image of a cross‐section of the pristine oxide showing the densely‐packed arrangement of material. The inset shows a Fast‐Fourier transform (FFT) diffractogram revealing the reflections characteristic of the Co_3_O_4_ spinel structure. e) A high‐resolution TEM image and its FFT diffractogram confirming the crystal structure of the Co_3_O_4_ spinel phase. Additional data is presented in Video S1 (Supporting Information).

The catalyst was prepared in situ by oxidizing a metallic cobalt foil (**Figure** **1**a) with oxygen at 615 °C for 1 h in our OSEM reactor.^[^ ^30^ ^]^ This treatment induced significant morphological changes (Figure 1b and Video S1, Supporting Information), corresponding to the formation of a dense overlayer of an intertwined material at the surface of the metallic substrate. A closer look at the surface of this overlayer (Figure 1c) reveals an intricate structure of interconnected grains. An analysis of its internal structure achieved by TEM imaging of a thin lamella (Figure 1d) untangles a densely packed material of fused and intergrown crystals seemingly without any significant interstitial pores. A Fast‐Fourier transform (FFT) analysis of the TEM image (see inset in Figure 1d) and high‐resolution TEM imaging of this overlayer (Figure 1e) evidence a crystalline material corresponding to the structure of the Co_3_O_4_ spinel phase.^[^ ^35^ ^]^ A selected‐area electron‐diffraction (SAED) analysis (Figure S1, Supporting Information) confirms the formation of the spinel.

### 2.2 Gas‐Phase 2‐Propanol Oxidation in Dry Feed

**Figure 2:**
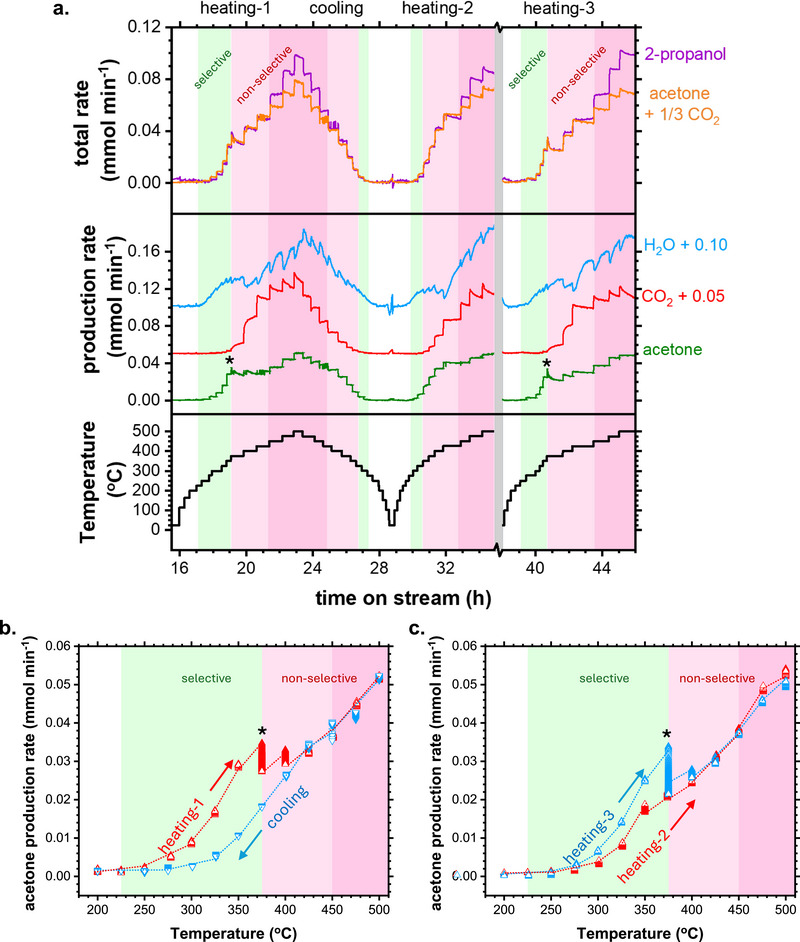
Catalytic performance of Co_3_O_4_ catalyst during gas‐phase 2‐propanol oxidation in a dry feed. a) Time‐resolved production rates of acetone (green), CO_2_ (red), H_2_O (blue), and a comparison between the conversion rate of 2‐propanol (purple) and the combined production rates of acetone and CO_2_ (orange). The temperature profile is shown in black, featuring heating and cooling intervals. Time break between 35 and 38 h represents a reoxidation step after the second heating treatment and the subsequent stabilization time. b) Production rate of acetone during heating‐1 and cooling cycles as a function of temperature. c) Production rate of acetone during heating‐2 and heating‐3 cycles as a function of temperature. The markers (*) denote transition regimes of fast acetone formation followed by deactivation. Green regions denote selective acetone formation regimes. Pink regions denote combined acetone and CO_2_ formation completing the carbon balance of 2‐propanol. Dark pink regions denote combined acetone and CO_2_ formation not completing the carbon balance of 2‐propanol. Data acquired at 50 Pa in 3N mL min^−1^ 2‐propanol: 3N mL min^−1^ O_2_: 3N mL min^−1^ Ar over oxidized Co foil (initial mass=71.2 mg). Additional data is presented in Figure S1 (Supporting Information).

The prepared catalyst was cooled down to room temperature and subsequently exposed to the reaction feed containing 2‐propanol vapor and O_2_. **Figure** **2**a shows the time series of the catalytic data obtained during the experiment. The Figure presents the heating protocol and the conversion rates of acetone, water, and CO_2_. It also includes the total rate of 2‐propanol conversion and the sum of acetone and CO_2_ production rates according to the reaction stoichiometries (Table 1). Additional data are provided in Figure S1 (Supporting Information).

Initially, the catalyst was heated stepwise to 500 °C (heating‐1), then cooled to room temperature (cooling), and subsequently reheated to 500 °C (heating‐2). Afterwards, the catalyst was reoxidized in an atmosphere containing O_2_ at 615 °C, following the same procedure of the preparation of the fresh material. This step is indicated by a vertical break in Figure 2a at time on stream (TOS) between 35 and 38 h. Subsequently, the catalyst was cooled back to room temperature and reheated stepwise to 500 °C under the reaction feed (heating‐3).

During heating‐1, acetone and water were produced without CO_2_ formation at temperatures between 225 and 375 °C, indicating a regime of selective oxidation (green regions). With further heating between 375 and 425 °C, CO_2_ was also detected, indicating a regime with total oxidation activity (pink regions). In the kinetic transition between the selective and the total oxidation regimes, the acetone production rate reached a maximum and subsequently decreased (see * symbol). The carbon balance between 2‐propanol, CO_2_ and acetone was strictly verified up to this point. At temperatures above 425 °C, CO_2_ formation continued to increase with each temperature step, although the reaction trace shows an initial boost followed by gradual deactivation. Water showed a complex behavior in this regime, with an initial decrease followed by a steep increase and subsequent stabilization upon each temperature step. In this regime, indicated by a dark pink color in Figure 2a, the carbon balance was not achieved, implying the transformation of 2‐propanol into products not accounted for in Table 1. Note, propylene was not produced at any temperature (Figure S1, Supporting Information).

The product traces followed decreasing trends during the cooling steps, and increasing trends during the heating‐2 treatment. Notably, the regimes of selective acetone formation were significantly diminished. During cooling, acetone was selectively produced only between 325 and 300 °C, with a formation rate of just ≈17% compared to the activity observed at this temperature range during the first heating. Similarly, during heating‐2, the selective regime was observed only between 325 and 350 °C, with conversion rates reaching only ≈39% compared to the values observed during heating‐1 at the same temperatures. In brief, the catalyst ability for selective oxidation was severely hindered after the first heating treatment.

The selective regime of heating‐1 was reestablished during heating‐3, as shown by the increasing acetone production rate upon the heating steps between 225 and 375 °C, in which the acetone trace ultimately peaked in a maximum (* symbol) and deactivated with concomitant CO_2_ activation. Hence, the catalytic features observed during the first heating were recovered after the reoxidation treatment. To get an idea, Figure 2b,c present the acetone production rate as a function of temperature during the successive treatments. The plots clearly denote differences in acetone production depending on the catalyst history. For instance, the production rate at 325 °C before reaching the deactivation regime, was 5.5x higher during the first heating than during the cooling. Furthermore, the data show that the reoxidation treatment was beneficial for recovering the selective regime of acetone formation, since the production profiles were almost identical during the first and the third heating, and the production rates reached similar values (compare Figure 2b,c).

**Figure 3:**
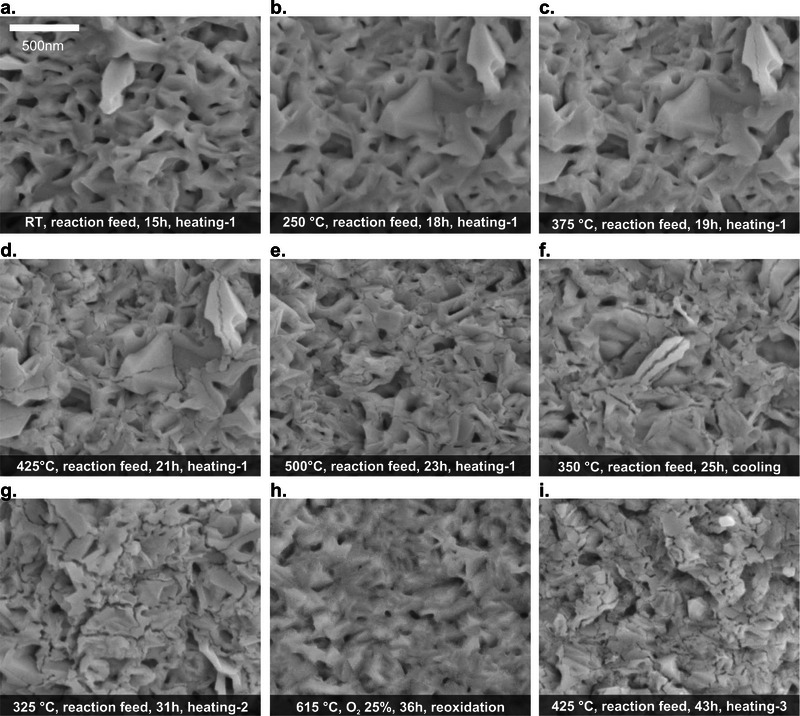
A compilation of in situ SEM images showing the morphological evolution of the catalyst surface during the gas‐phase 2‐propanol oxidation in dry feed. a) Image of the catalyst surface at room temperature in the reaction feed. b) Image of the catalyst surface in the reaction feed at 250 °C during the first heating. c) Image of the catalyst surface in the reaction feed at 375 °C during the first heating, revealing cracks at the oxide. d) Image of the catalyst surface in the reaction feed at 425 °C during the first heating, revealing increasingly prominent cracks. e) Image of the catalyst surface in the reaction feed at 500 °C during the first heating, showing fragmented and heterogeneous surface morphologies. f) Image of the catalyst surface in the reaction feed at 350 °C during the cooling, showing that the cracks have remained at the oxide. g) Image of the catalyst surface in the reaction feed at 325 °C during the second heating. h) Image of the catalyst surface during reoxidation in 25%O_2_ atmosphere at 615 °C, showing that the cracks were refilled. i) Image of the catalyst surface in the reaction feed at 425 °C during the third heating showing that the cracks have reappeared at the oxide. Data acquired at 50 Pa in 3N mL min^−1^ 2‐propanol: 3N mL min^−1^ O_2_: 3N mL min^−1^ Ar. Additional data is presented in Videos S2–S6 (Supporting Information).

To understand these observations, we examined the catalyst surface by in situ SEM imaging. **Figure** **3**a shows that the oxide overlayer did not undergo any appreciable change by the presence of the reaction feed at room temperature (compare with Figure 1b). Furthermore, the oxide layer remained during heating‐1 (Figure 3b–e and Video S2, Supporting Information) seemingly without any alterations, up to 375 °C (Figure 3b,c). At this point, nanometric cracks started to develop on the oxide crystals (Figure 3c), although the crystalline morphology of the overlayer material was still preserved. These cracks became larger and more abundant with increasing temperatures (Figure 3d) and TOS, giving rise to a highly heterogeneous surface (Figure 3e). The cracks remained on the catalyst during the cooling (Figure 3f and Video S3, Supporting Information) and the second heating cycles (Figure 3g and Video S4, Supporting Information).

Notably, the surface was partially “healed” after the reoxidation treatment (Figure 3h and Video S5, Supporting Information), involving a refilling of the cracks and the smoothening of the remaining surface, although the morphology of the pristine oxide was not restored. Afterwards, the cracks reappeared on the catalyst when the system was heated for the third time in the reaction feed at temperatures above 375 °C (Figure 3i; Figure S2 and Video S6, Supporting Information).

In brief, the combined OSEM data show that the 2‐propanol oxidation occurred in three well‐defined regimes: a low‐temperature regime ranging from 225 to 375 °C characteristic for selective acetone formation, a high‐temperature non‐selective regime, in which also CO_2_ was formed,^[^ ^11^, ^25^ ^]^ and a regime at very high temperatures (≈450 °C), where other degradative reactions occurred, probably indicating molecular fragmentation or coking. Once the temperature surpassed the selective oxidation regime, the surface structure transitioned into a cracked material. The reoxidation treatment restored the catalytic behavior and healed the cracked surface, indicating a strong correlation between the morphological characteristics of the catalyst and its performance.

**Figure 4:**
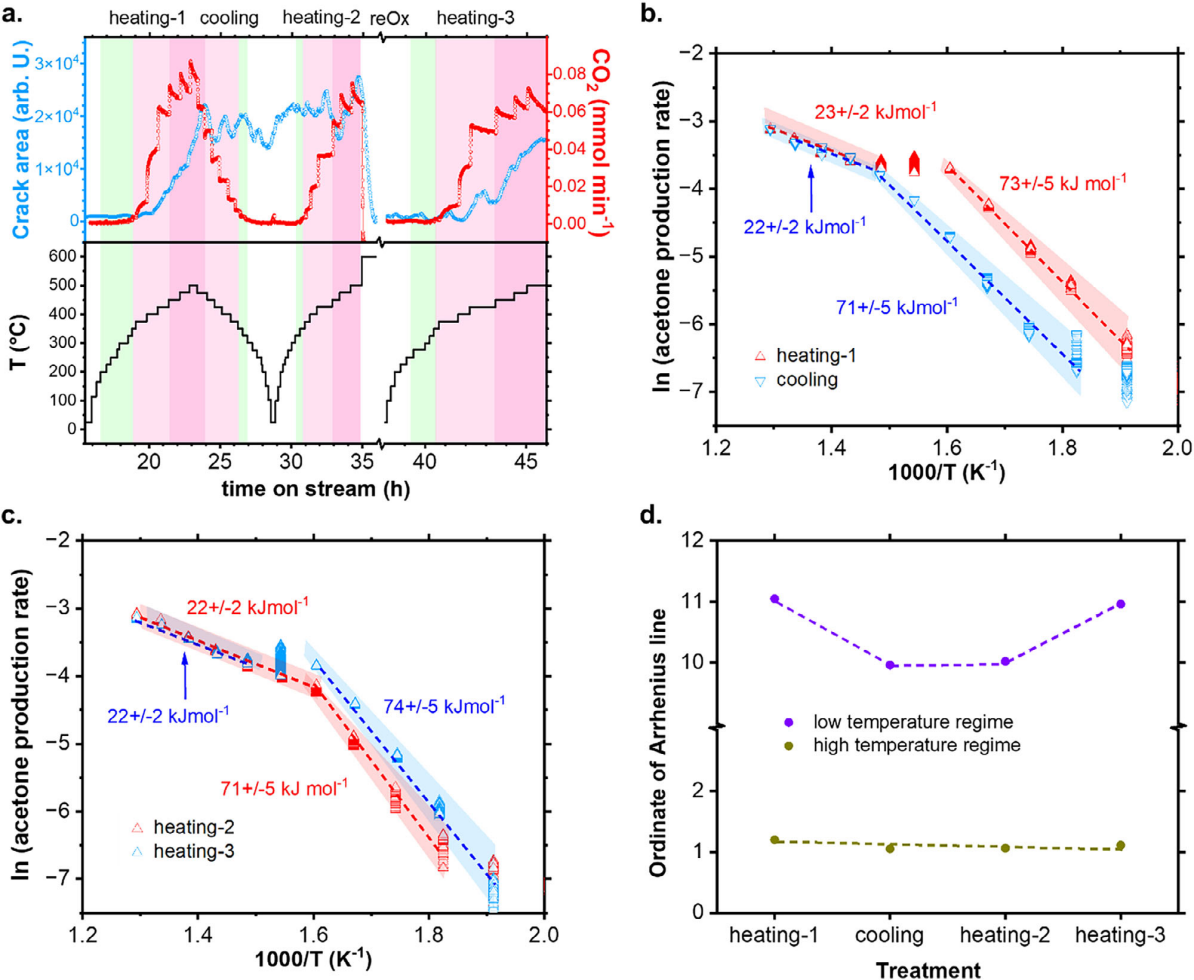
Correlation between catalyst morphology, reaction selectivity and activation parameters during the catalytic reaction in a dry feed. a) A comparison between the surface area representative of the cracks and the formation of CO_2_ during the successive treatments.The time axis break between 35 and 37 h represents the reoxidation (ReO_x_) step after the second heating treatment and the subsequent stabilization time. The green regions represent the regimes of selective acetone formation. Additional data is presented in Video S7 (Supporting Information). b) Arrhenius plots of catalyticacetone formation during the first heating and the cooling stages. c) Arrhenius plots of catalytic acetone formation during the second and the third heating stages. d) Evolution of the ordinate of the linear Arrhenius trends during the consecutive treatments.

To explore this correlation, we developed an automatized SEM image segmentation model that determines the extents of crack formation. By training a U‐NET with LSTM block with synthetic data, the model can reliably segment the observed crack features over the complex morphology of the oxide overlayer. The technical details and validation of this model can be consulted in our previous work,^[^ ^31^ ^]^ in which we used the same reaction and catalyst material. The results (**Figure** **4**a and Video S7, Supporting Information) show that the SEM image area representative of the cracks remained close to the base level during the selective oxidation regime, at temperatures below 375 °C. The area of the cracks increased once the system surpassed 375 °C, coinciding with the activation of CO_2_ formation. The crack area stabilized during the subsequent cooling and heating‐2 treatments. The reoxidation treatment induced the refilling of the cracks (see Figure 3h), which is observed in Figure 4a as a steep drop of the crack area back to the base level, at TOS═35 h. This quantity remained close to zero at TOS between 38 and 41 h, at temperatures below 375 °C, only to increase again during the kinetic transition of heating‐3, coinciding with the activation of CO_2_ formation. Hence, the initiation of crack formation seems to signal the onset of CO_2_ formation and acetone deactivation. Furthermore, the presence of cracks consistently indicates low acetone selectivity across the temperature range.

We also applied an Arrhenius analysis to the catalytic data focusing on the acetone formation. For the first heating (Figure 4b, red data points), the plot shows a linear increase during the selective regime, with an apparent activation energy (*E_app_*) of 73 kJ mol^−1^. With increasing temperatures, the plot evinces the transition from the selective into the non‐selective regimes, in which the *E_app_* of acetone formation was found to be 23 kJ mol^−1^. Hence, the measured changes in the *E_app_* values correlate to the kinetic transition and the catalyst morphological transformation, probably due to changes in the mechanism of acetone formation or the occurrence of degradative reactions. During the cooling stage (Figure 4b, blue data points) the activation energies remained virtually unaltered for both the non‐selective (22 kJ mol^−1^) and the selective (71 kJ mol^−1^) regimes. Similar activation energies were measured during the second and the third heating cycles (Figure 4c), corresponding to 71–74 kJ mol^−1^ for the selective regimes, and 22 kJ mol^−1^ for the non‐selective regimes, respectively.

Complementarily, it can be seen from the Arrhenius plots that the height of the linear trends changed during the successive treatments. We represent this information in Figure 4d. For the selective regime, the ordinates were higher during the first heating compared to the subsequent cooling and second heating treatments. This value increased again during the third heating, indicating a recovery after reoxidation. The values for the non‐selective regime remained steady during the different treatments.

**Figure 5:**
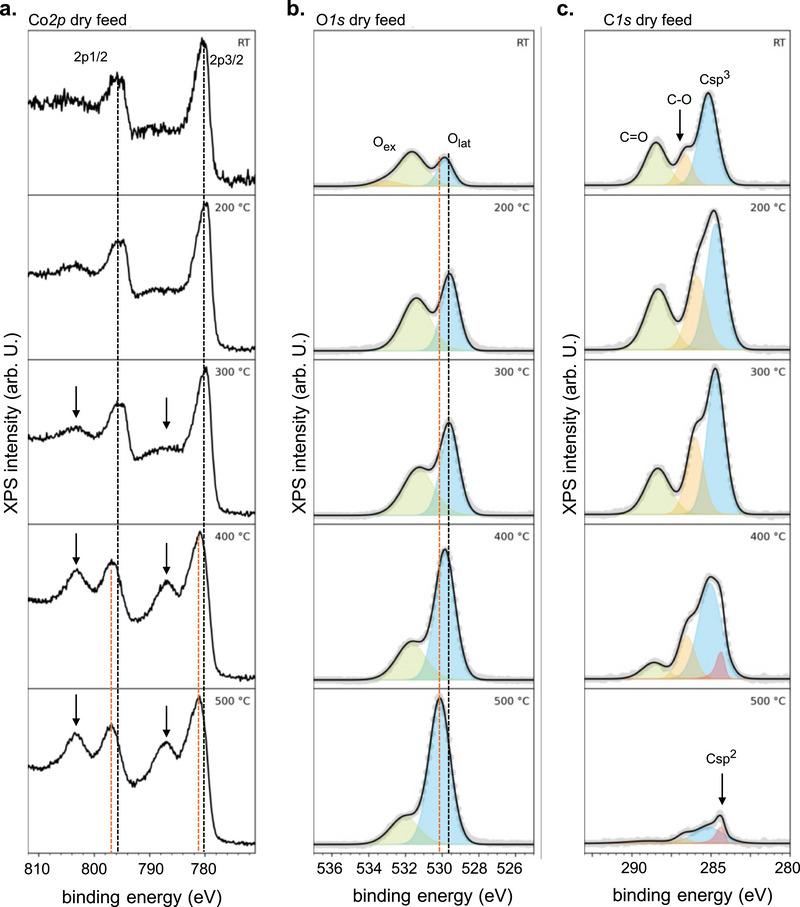
NAP‐XPS spectra showing the evolution of the catalyst surface species during 2‐propanol oxidation in a dry feed as a function of temperature. a) NAP‐XPS Co2p spectra at increasing temperatures (top to bottom). The dotted black lines mark the initial positions of the 2p3/2 and 2p1/2 signals at room temperature. The dotted orange lines mark the final positions of the 2p3/2 and 2p1/2 signals at 500 °C. The arrows mark the emergence of satellite signals. b) NAP‐XPS O1s spectra at increasing temperatures (top to bottom). c) NAP‐XPS C1s spectra at increasing temperatures (top to bottom). Data acquired at 50 Pa in 3Nccmin^−1^ 2‐propanol: 3Nccmin^−1^ O_2_: 3Nccmin^−1^ Ar. Additional data is presented in Figure S4 (Supporting Information).

To understand the catalyst evolution under reaction conditions, we characterized the system by NAP‐XPS experiments. At room temperature (**Figure** **5**a), the XP spectra of Co*2p* show the characteristic signals of the spinel phase, represented by the *2p_3/2_* peak position at binding energy (BE) 779.6 eV, the *2p_1/2_* peak at 794.8 eV, and a relatively flat shape of the satellite signals in the regions between 785–789 eV and 802–806 eV, respectively.^[^ ^24^, ^36^ ^]^ The spinel structure was seemingly preserved upon heating to 300 °C. At higher temperatures, the *2p_3/2_* and *2p_1/2_* signals shifted into higher binding energies (see orange lines), and distinctive satellite peaks appeared in the spectra (see arrows in Figure 5a). These results point to the reduction of the spinel surface into CoO_x_ phases.^[^ ^24^, ^36^ ^]^

The O*1s* spectra (Figure 5b) show the typical contributions of lattice oxygen (O_lat_) species (529.5–530.5 eV),^[^ ^24^ ^]^ and adsorbed oxygen species, “O_ex_”, at 531.5–532.5 eV,. The latter has been ascribed to adsorbed atomic oxygen, adsorbed molecular oxygen, and (oxy)hydroxides.^[^ ^37^, ^38^ ^]^ The data show that the contribution of these O_ex_ species decreased with each temperature increment, implying a consumption of the adsorbed oxygen during the reaction. Furthermore, the position of the O_lat_ signal was found to be shifting to higher BE at temperatures above 400 °C (see orange line). The trends of the O*1s* spectra were reversed when the catalyst was subsequently cooled stepwise from 500 °C to room temperature in the reaction feed (Figure S3, Supporting Information).

The C*1s* spectra (Figure 5c) show the three typical components previously observed in 2‐propanol adsorption experiments at room temperature.^[^ ^24^ ^]^ The peaks are centered at 284.6, 286.4, and 288.3 eV, and can be attributed to C‐C, C‐O, and carbonyl (C═O) functionalization's, respectively.^[^ ^24^ ^]^ This result suggests the presence of C═O already at room temperature. The C*1s* signals remained in the spectra at temperatures up to 300 °C. However, their relative areas changed during this treatment, as the carbonyl signal became less intense compared to the C‐O contribution. At 400 °C, the absolute intensity of the spectrum was diminished, and the carbonyl signal became even less intense compared to the other contributions. Also, species containing C═C bonds started to be detected (BE═ 283.7 eV). The spectral intensity further decreased at 500 °C. Only a minor contribution of C‐C species was detected at this temperature, while the relative contribution of C═C became more dominant. This observation may indicate the presence of solid carbon or graphitized deposits at the highest temperatures of the experiment. These changes in the C*1s* spectra were reversed when the catalyst was subsequently cooled from 500 °C to room temperature in the reaction feed (Figure S4, Supporting Information).

In brief, the NAP‐XPS data show a definite change in the surface species coinciding with the temperature regime of the kinetic transition detected in the OSEM experiment. The catalyst surface was reduced from spinel into rock‐salt (CoO_x_) during this transition.

### 2.3 Gas‐Phase 2‐Propanol Oxidation in Wet Feed

**Figure 6:**
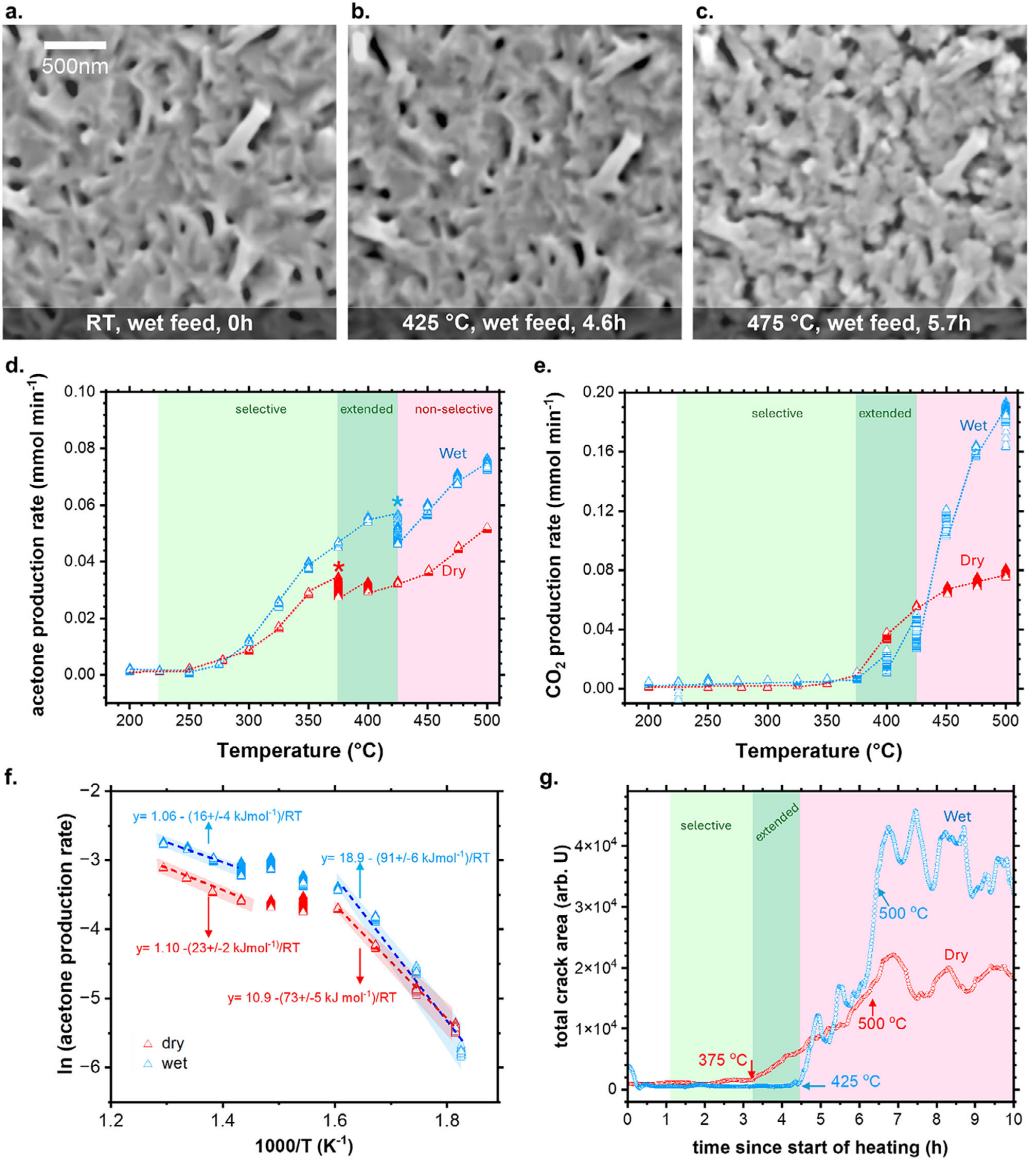
A comparison between surface evolution and reaction performance during gas‐phase 2‐propanol oxidation in wet and dry feeds. a) In‐situ SEM image of the catalyst surface at RT revealing the preservation of the oxide material in wet feed. b) In‐situ SEM image of the catalyst surface at 425 °C in wet feed, revealing preservation of the oxide layer. c) In‐situ SEM image of the catalyst surface at 475 °C in wet feed, revealing crack formation and surface fragmentation. d) A comparison between the production rate of acetone in wet and dry (heating‐1) feeds as a function of temperature. The (*) symbol marks the end of the selective regime followed by acetone deactivation. e) A comparison between the production rate of CO_2_ in wet and dry (heating‐1) feeds as a function of temperature. f) A comparison of the acetone production rate in Arrhenius space in wet and dry (heating‐1) feeds. g) A comparison between the surface area representative of the cracks in wet and dry (heating‐1) feeds as a function of the treatment duration. Green regions denote regimes of selective acetone formation in wet and dry feeds. Dark green regions denote extended regimes of selective acetone formation only in wet feed. Pink regions denote intense degradation of 2‐propanol in both feeds. Data acquired at 62 Pa in 3NmLmin^−1^ 2‐propanol: 3NmLmin^−1^ O_2_: 1Nccmin^−1^ Ar: 2NmLmin^−1^ H_2_O. Additional data is presented in Videos S8 and S9 (Supporting Information).

Next, we investigated the effect of co‐feeding water vapor on the reaction under identical gas velocities and reactant compositions. The results are compiled in **Figure** **6**, Figure S5 and Video S8 (Supporting Information). An inspection to the catalyst surface by in situ SEM imaging corroborated the presence of the oxide crystals at the beginning of the reaction (Figure 6a and Video S8, Supporting Information), and their preservation upon heating (Figure 6b and Video S8, Supporting Information) up to 425 °C. However, this oxide layer collapsed rapidly once the temperature surpassed 425 °C, yielding a highly fragmented material with a high concentration of cracks and voids (Figure 6c and Video S8, Supporting Information).

These morphological changes correlated to variations in catalytic activity. For instance, the acetone production rate increased with each temperature step between 225 and 375 °C (Figure 6d, green region). Notably, the formation of acetone continued to increase up to 425 °C (Figure 6d, dark green region). For comparison, the data of acetone formation during the first heating experiment in dry feed is also presented in Figure 6d, revealing an extended window of selective acetone formation with increased formation rates (≈80%) when water vapor was introduced into the feed (see dark green regions). In addition, the CO_2_ formation rate remained lower in the wet feed compared to the dry feed experiment during this extended window (Figure 6e). However, at temperatures above 425 °C, the CO_2_ formation was dramatically potentiated in the wet feed (pink region of Figure 6e), giving rise to an increase of ≈110% compared to the dry feed reaction at 500 °C.

Complementarily, the *E_app_* values of acetone formation in wet feed were 91 kJ mol^−1^ for the selective regime, and 16 kJ mol^−1^ for the degradative regime (Figure 6f). The value of the ordinate in the Arrhenius space was much higher in the wet feed compared to the value measured during the first heating experiment in dry feed.

We also investigated the correlation between the surface morphology and the catalytic function in the wet feed treatment following the extent of crack formation (Figure 6g and Video S9, Supporting Information). For comparison, we present the data of the first 10 h of the dry feed experiment, starting from the initiation of the reaction. At times between 0 and 3.3 h corresponding to temperatures below 375 °C, the area of the cracks remained close to the base level (light green region) in both wet and dry feeds. Above this temperature, the crack area increased for the dry feed experiment but remained at the base level for the wet feed run up to 425 °C (dark green region). Once the temperature surpassed 425 °C, the crack area increased steeply for the system in the wet feed, coinciding with the pronounced activation of CO_2_ production (Figure 6e). The final area of the cracks reached higher values in wet than in dry feed, reflecting a larger number of cracks and surface heterogeneities after the kinetic transition.

In brief, the addition of water in the feed enhanced the acetone production and extended the dynamic window for selective oxidation. However, it potentiated the formation of cracks, fragmented material, and CO_2_ once the system reached excessively high temperatures.

**Figure 7:**
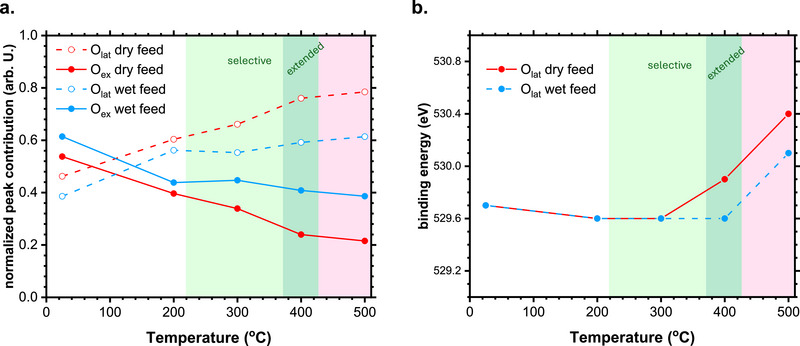
The evolution of the catalyst oxygen species depends on the reaction conditions. a) A comparison between the relative peak contributions of lattice oxygen (O_lat_) and adsorbed oxygen (O_ex_) species as a function of temperature in wet and dry feeds. b) Position of the O_lat_ peak as a function of temperature in wet and dry feeds.

These phenomena were also investigated by NAP‐XPS experiments in the wet feed (Figures S6 and S7, Supporting Information). The O*1s* spectra (Figure S6, Supporting Information) show the presence of O_lat_ (BE═ 529.5–530.5 eV) and O_ex_ (BE═ 531.5–532.5 eV) species. Although the intensity of the O_ex_ signal also decreased with increasing temperatures as in the dry feed experiment, the peak was still relatively large even at 500 °C. For comparison, we present in **Figure** **7**a the relative peak contributions of the O*1s* signals in wet and dry feeds. Clearly, the O_ex_ signal decreased with increasing temperatures, although this effect was less marked in the wet environment, suggesting a stabilizing influence of the water co‐feed on the catalyst oxygen. We also plotted the O_lat_ peak position depending on the reaction temperature (Figure 7b). The Figure shows that the BE of the O_lat_ peak was stable up to 300 °C in both feeds. At 400 °C, this peak upshifted in the dry feed experiment, coinciding with the kinetic transition and surface morphological evolution in this environment. However, the peak position remained steady up to 400 °C in the wet feed. It moved to upshifted BE only at 500 °C, i.e., after the kinetic transition in this environment. Because the BE value is the difference between the electronic state and the Fermi level, a shift in the peak position often implies that the Fermi level has changed,^[^ ^39^, ^40^, ^41^ ^]^ which is an indication of changes in the catalyst redox state. In brief, the O_lat_ peak position correlates to the overall regime of acetone formation and surface morphology.^[^ ^41^ ^]^ in both environments, with upshifted peak positions indicating the kinetic transition towards low acetone selectivity.

The C*1s* signals revealed the same species in wet and dry feeds (Figure S7, Supporting Information), with the general decrease of the total carbon signal and the vanishing of the C═O contribution at temperatures ≈300 °C. A very strong signal of C═C carbon species (283.7 eV) dominated the spectra at temperatures above 400 °C.

### 2.4 Post Catalytic Characterization

**Figure 8:**
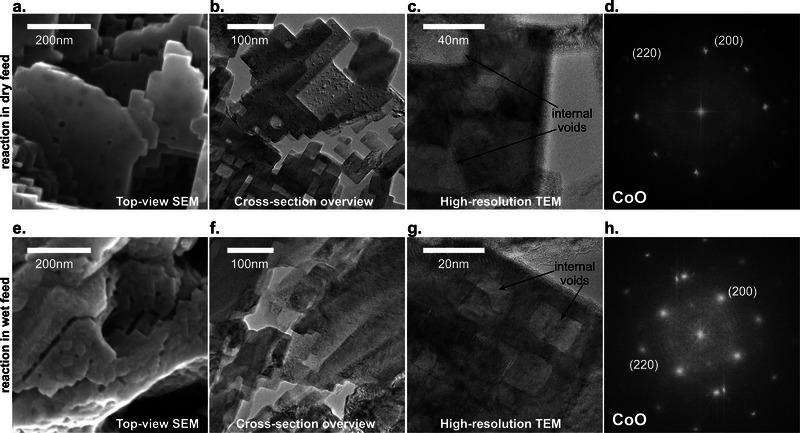
Electron microscopy characterization of the spent catalysts. a) An SEM image of the catalyst surface after 2‐propanol oxidation in dry feed revealing a rectangular surface morphology. b) A TEM (bright‐field) image of a cross‐section of the catalyst after reaction in dry feed showing the cubic‐like morphology of the internal material and the presence of serrated interstitial openings. c) A TEM (bright‐field) image of a cubic‐like particle showing the presence of rectangular voids within the structure. d) A fast‐Fourier transform (FFT) diffractogram of the image in c) revealing the typical reflections of rock‐salt CoO phase. e) An SEM image of the catalyst surface after 2‐propanol oxidation in wet feed revealing a rectangular surface morphology. f) A TEM (bright‐field) image of a cross‐section of the catalyst after reaction in wet feed showing the cubic‐like morphology of the internal material and the presence of interstitial serrated openings. g) A TEM (bright‐field) image showing the presence of rectangular voids within the particles. h) A fast‐Fourier transform (FFT) diffractogram of the image in g) revealing the typical reflections of rock‐salt CoO phase.

**Figure** **8** depicts a compilation of electron microscopy images of the post‐reaction materials. The spent sample recovered from the dry feed experiment (Figure 8a) shows an arrangement of rectangular particles that contrast with the pristine surface morphology obtained by oxidation of the metallic foil (Figure 1b,c). An inspection to the internal structure of this overlayer (lamella, Figure 8b) reveals the prevalence of the rectangular morphology also at the inner layers of the catalyst. This internal arrangement is characterized by interparticle openings and interstices of serrated texture. Furthermore, the material exhibits internal voids within the oxide that retain a cubic‐like geometry (Figure 8b,c). We determined the presence of crystalline phases in the rectangular particles by a Fast‐Fourier transform (FFT) analysis of Figure 8c, showing the typical reflections of the rock‐salt structure of CoO_x_ (Figure 8d). We confirmed this result by SAED analysis of the crystalline material,^[^ ^35^, ^42^ ^]^ (Figure S8, Supporting Information).

Figure 8e also reveals the rectangular morphology of the spent catalyst recovered from the reaction in the wet feed, although the surface structure appears more irregular compared to the sample of the dry feed experiment (see Figure 8a). The rectangular motifs (Figure 8f) also occurred at the internal structure of the catalyst. Furthermore, the cubic particles also exhibited internal voids retaining a cubic‐like geometry (Figure 8g). The FFT analysis of this image (Figure 8h) and the SAED pattern (Figure S9, Supporting Information) analysis of this sample also show reflections characteristic of the cubic CoO_x_ phase.^[^ ^35^, ^42^ ^]^

In brief, the reaction transformed the spinel catalyst into rock‐salt. The formation of the serrated texture observed by TEM imaging can be correlated to the cracks detected in situ at lower lateral resolution in the OSEM experiments. Hence, the formation of these cracks seems to indicate the bulk transformation of the spinel oxide into rock‐salt. This latter phase contained internal defects, notably cubic‐like voids that may imply a mechanism of vacancy/point defect migration.

### 2.5 Ab Initio Thermodynamics and Vacancy Transport in Bulk CoO and Co_3_O_4_

We furthered our study with a theoretical approach to the effect of water vapor on the catalytic system by ab initio thermodynamics considerations at conditions mimicking the OSEM experiments. The details can be consulted in the second section of the supporting material. For these calculations, we referenced the O_2_ chemical potential to the standard free energy of the water formation reaction. The calculations at isothermal steps between 375 and 500 °C show that the absolute oxygen potential expressed per O atom was always larger in the wet feed compared to the dry feed under the experimental conditions. Furthermore, this difference was found to increase from 0.549 eV at 375 °C to 0.654 eV (lowest bound) at 500 °C. In terms, these results mean that any formation of oxygen vacancies is suppressed by a factor of 10^5^ to 10^6^ at 375 °C due to the water vapor presence in the reaction stream.

We also calculated the free energy of formation of oxygen vacancies in both CoO and Co_3_O_4_ using density functional theory (DFT) calculations, and estimated their migration kinetic barriers in these phases by Climbing‐Image Nudged Elastic Band (CI‐NEB). The free energy of formation was found to be 0.226 eV higher in the spinel than in the rock‐salt, implying a phase‐intrinsic higher thermodynamic cost in the spinel for vacancy formation. In addition, the kinetic barrier of vacancy migration into the bulk following a nearest‐neighbor hop mechanism was found to be 2.93 eV in the spinel, and 1.62 eV in the rock‐salt. These values imply a spinel/rock‐salt diffusivity ratio of 10^−9^ to 10^−11^ at the experimental temperature range.

In brief, any created vacancies migrate much faster in the rock‐salt than in the spinel. With the water co‐feeding acting as an oxygen buffer that raises the oxygen potential and the free energy required to induce vacancies in the catalyst, the combined effects delay the spinel‐to‐rock‐salt transition and potentiate the availability of active oxygen species in the wet feed.

## 3 Discussion

Our results confirm previous studies showing that the gas‐phase 2‐propanol oxidation in dry conditions occurs in distinct regimes over Co_3_O_4_ spinel catalysts.^[^ ^11^, ^24^, ^25^, ^27^ ^]^ At low temperatures (up to 375 °C) acetone is the only product, while at high temperatures (375–500 °C) CO_2_ is also produced. The transition between the selective and the non‐selective, degradative regimes was marked by a catalyst transformation from the spinel phase into rock‐salt. According to our NAP‐XPS results (Figure 5 and Figure 7a), the content of adsorbed O_ex_ species decreased with increasing temperatures, and C═O signals were already observed at room temperature. These observations indicate that the formation of acetone may occur by reaction of 2‐propanol with adsorbed O_ex_ species, pointing to a surface reaction mechanism similar to a Langmuir‐Hinshelwood kinetics.^[^ ^27^ ^]^ As proposed earlier,^[^ ^11^, ^43^ ^]^ due to these adsorbed oxygen species the acetone formation may follow a dominant Langmuir‐Hinshelwood mechanism at the earlier stages of the reaction when the temperature is still low. This possibility has been substantiated by 2‐propanol programmed temperature desorption and decomposition experiments on the spinel, showing the evolution of acetone even without O_2_ co‐feeding at temperatures slightly above room temperature.^[^ ^11^, ^44^ ^]^ Furthermore, the cobalt oxide spinel is known for its ability to dissociatively adsorb oxygen,^[^ ^45^ ^]^ which could indicate a catalytic path favoring the surface reaction of 2‐propanol with these dissociated species. With increasing temperature, the catalytic rate is increased, which explains the consumption of O_ex_ species, as shown in our XPS data (Figure 7a).

However, surface activation as the only path for acetone formation cannot explain all the observed structural phenomena. For instance, while the fraction of O_ex_ species dropped significantly when the system approached the kinetic transition, the BE of the O_lat_ species started to shift upwards (Figure 7b). This result implies the emergence of electrostatic imbalances in the catalyst bulk compared to the pristine structure, for instance, due to partial reduction and creation of defects,^[^ ^39^ ^]^ which are not expected in surface activation alone. It has previously been shown that the redox speciation of the Co_3_O_4_ has a marked influence on the catalytic behavior during CH_4_^[^ ^46^ ^]^ and CO^[^ ^47^ ^]^ oxidation, where a Mars van Krevelen mechanism has been proposed as a secondary path to the reaction kinetics. Similarly, the acetone formation could imply a parallel MvK path that becomes dominant with increasing temperatures. This alternate path can explain the upshift of the O_lat_ signal when the system approaches the kinetic transition, which could be interpreted as the creation of vacancies in the structure due to the consumption of lattice oxygen, inducing electrostatic effects in the catalyst.^[^ ^39^, ^40^, ^41^, ^47^ ^]^ Our combined results in dry feed (Figures 2, 3, 4, 5 and 8a) also show that the pristine Co_3_O_4_ structure was transformed into a highly cracked, defective material containing rock‐salt CoO_x_ and internal voids after the reaction cycles (Figure 3, Figure 4a and Figure 8b; Figures S6 and S7, Supporting Information), confirming the catalyst reduction by the chemical environment and the loss of lattice oxygen.

The occurrence of the cracks detected in the OSEM imaging is a manifestation of this catalyst reduction, as expected from the bulk properties of the spinel and rock‐salt structures. For instance, the densities at room temperature are 6.10 g cm^−3 [^ ^48^ ^]^ and 6.66 g cm^−3^^[^ ^49^ ^]^ for the spinel and the rock‐salt structures, respectively. Consequently, the reductive phase transformation involves a volumetric contraction that, if not spatially homogeneous, induces internal strain fields that can lead to structural defects such as dislocations, grain boundary distortions, and microcracks.^[^ ^50^ ^]^ These defects arise because the differential shrinkage at the contracting phase cannot be fully accommodated elastically at the crystal lattice. Hence, the cracks of the OSEM experiment reveal the reaction‐induced phase transition, indicating that the system has left the frustrated regime of high catalytic performance, and enters the regime of reduced acetone selectivity (Figure 2). In addition to the phase transformation, we found cubic‐like voids within the oxide (Figure 8). All these results corroborate that a mechanism of lattice oxygen consumption and vacancy creation was operative during the catalytic process,^[^ ^32^, ^47^, ^51^ ^]^ in agreement with MvK dynamics.

**Figure 9:**
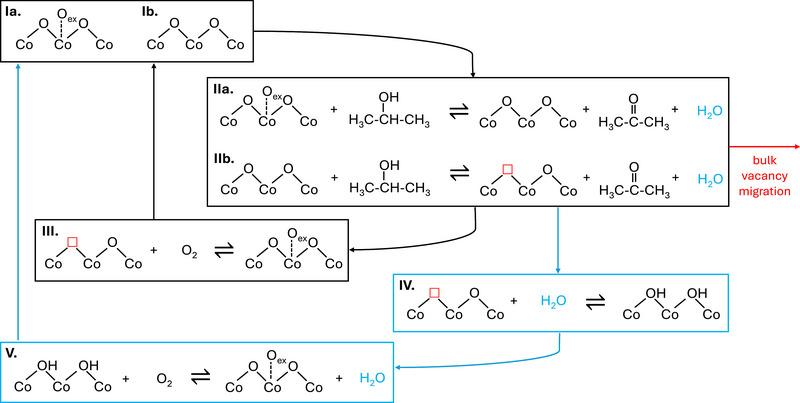
Schematized mechanistic steps of 2‐propanol oxidation over Co_3_O_4_ illustrating the interplay between surface and lattice oxygen species in dry and wet feeds. Steps IIa and IIb represent 2‐propanol oxidation via surface O_ex_ and lattice O_lat_ species, producing acetone and water. Path IIb generates oxygen vacancies (see red □ symbol). The vacancies are either replenished by O_2_ (Step III) or migrate into the bulk, triggering irreversible structural changes (see red arrow). Under wet conditions, water assists in refilling vacancies through hydroxylation (Step IV) and subsequent reoxidation by O_2_ (Step V), thereby stabilizing the catalyst surface and maintaining the active spinel phase. Blue arrows represent water‐assisted recovery paths.

With this information, we think that the formation of acetone in dry feed can be explained by the steps schematically depicted in **Figure** **9**. Initially, the spinel surface exhibits both O_ex_ (Ia) and O_lat_ (Ib) species that can activate the 2‐propanol. Accordingly, the reaction occurs by either consumption of the O_ex_ surface species (step IIa), namely the surface activation path, or by reaction with lattice oxygen (step IIb), i.e., by MvK mechanism, producing an oxygen vacancy. The cycle is closed when O_2_ from the stream reoxidizes the surface, which resupplies both O_ex_ and O_lat_ species (step III). As mentioned above, we think that both mechanisms coexist in the selective regime.

Molecularly, the reaction is thought to occur by adsorption of the 2‐propanol, forming an alkoxide intermediate on the Co(III) site of octahedral symmetry, probably favored by the geometric distortions.^[^ ^52^, ^53^ ^]^ This alkoxide is later transformed into acetone by H‐C_sec_ abstraction.^[^ ^52^ ^]^ This description is compatible with our C*1s* XPS results, showing the presence of these surface species during the selective regime (Figure 5c). It has been shown that the breaking of metal‐O bonds is more favorable on octahedral than on tetrahedral sites of the spinel,^[^ ^54^ ^]^ in line with our observation that the preservation of the spinel is crucial to acetone selectivity (Figure 2 and Figure 5). Hence, the formation of acetone is favored on the Co_3_O_4_ due to preferred adsorption sites and a facile breaking of the O_lat_ bonds contributing to the MvK path and overall activity.

At temperatures approaching the phase transition, several changes occur. For instance, the high rate of acetone formation and the activation of CO_2_ production deplete the oxygen species (Table 1). Furthermore, the surface vacancies created by the MvK path may become mobile and migrate into the bulk due to the thermal energy, furthering the structural change and the permanent reduction of the catalyst. We have recently observed the occurrence of vacancies within the spinel by *operando* TEM experiments, demonstrating the tendency of the system to create vacancies even under milder reaction conditions.^[^ ^28^ ^]^ This observation is compatible with several studies showing the formation of hollow cobalt oxide nanocrystals due to vacancy mobilization during redox synthesis,^[^ ^32^ ^]^ the observation of reversible spinel‐to‐rock‐salt phase transitions induced by an electron probe and mediated by vacancies,^[^ ^55^ ^]^ and the known ability of cobalt oxides as anionic oxide carriers during the operation of solid oxide fuel cells.^[^ ^45^ ^]^

These observations align with our theoretical analysis, which indicate that vacancies migrate much faster and are more likely to be formed in the rock‐salt than in the spinel phase. Hence, when the system initiates the phase transition, the rate of vacancy migration into the bulk may become disproportionately large, furthering the completion of the phase transformation. At sufficiently high rates, the vacancies can coalesce into the large cubic voids observed in our characterizations (Figure 8). This interpretation also explains previous results showing that small Co_3_O_4_ nanoparticles (9 nm) were less stable and active than larger nanoparticles (19 nm),^[^ ^27^ ^]^ since a large bulk volume could be important to withstand the structural stresses associated with vacancy injection and phase transformation.

In **Figure** **9**, the active system must continuously maintain a balance between oxidized and partially reduced states of the spinel, O_ex_ and O_lat_ species, filled and unfilled vacancies, and several adsorbates during the stages of the catalytic cycle. This dynamic regime aligns to the mixed configuration characteristic of the frustrated phase transition, in which the active phase does not fully transit into either direction due to the competing interactions. This frustrated regime results of a combination of Langmuir‐Hinshelwood and Mars‐van Krevelen dynamics, requiring a continual resupply of active oxygen species not only for the reaction, but also for the structural integrity of the material. Excessively high temperatures trigger the complete transition into the rock‐salt phase and the decline of the catalytic performance. Since the driving factors behind the completion of the phase transition are the depletion of oxygen and the migration of vacancies into the bulk, suppressing these effects is key for enhancing the catalytic performance.

We addressed this aspect by investigating the reaction in the wet feed. Water is known to exert a significant impact over oxygen vacancies in several oxidation systems, including CO oxidation on CuO/CeO_2_^[^ ^56^ ^]^ and Pd/TiO_2_ catalysts,^[^ ^57^ ^]^ adsorbed/dissociated species on MgO,^[^ ^58^ ^]^ o‐xylene oxidation on CeO_2_ nanocubes,^[^ ^59^ ^]^ toluene oxidation on MgO‐supported Pt single‐atom catalysts,^[^ ^60^ ^]^ VOCs oxidation catalysts,^[^ ^61^ ^]^ and ZrO_2_ materials.^[^ ^62^ ^]^ These studies point to a rapid refilling of the oxygen vacancies by water. In agreement with these observations, our NAP‐XPS data (Figure 7) and theoretical analysis show an increased oxygen potential and a significant suppression of vacancy formation in the wet feed, both of which contribute to the extended selectivity window and increased acetone formation.

Catalytically, the data shows an extension of the selective acetone regime up to 425 °C (Figure 6d,e), where the production rate was enhanced by ≈80% compared to the dry feed. This behavior can be understood by the rapid refilling of surface vacancies and the increase of active oxygen species by the action of water, which is demonstrated by the stabilization of the O_lat_ signal even at 400 °C (Figure 7b), the increased relative contribution of the O_ex_ species at high temperatures (Figure 7a), and the delayed transition detected in the OSEM (Figure 6a–c,g). The interaction of water with the vacancies can also explain the complex water trace in the dry feed experiments under non‐selective regimes (pink regions of Figure 2a), where the evolved water could be reacting in situ with the vacancies generated by the chemical potential.

We rationalize these results with the cycle I‐II‐IV‐V of Figure 9, involving similar steps in wet and dry feeds, up to the formation of the oxygen vacancies by MvK activation. In the wet feed, the vacancies are readily refilled by water, producing a momentary hydroxylation (step IV), which is followed by rapid reoxidation with gaseous O_2_ and water evolution ^[^ ^63^ ^]^ (step V), thereby resetting the system in the initial state (step I) before the vacancies migrate into the bulk. However, this positive influence cannot be sustained above 425 °C (Figure 6), as the accumulation of vacancies and defects rapidly degrades the catalyst structure, while the activation of CO_2_ formation furthers the depletion of active oxygen. These effects induce the complete phase transformation into rock‐salt. Because the phase transition in wet feed is kinetically delayed to higher temperatures, once initiated, it occurs very fast and leads to a highly heterogeneous material, as suggested by our computer vision analysis (Figure 6g) and corroborated by the electron microscopy characterizations (Figure 8e–h).

At the highest temperatures, the presence of C═C bonds in the C*1s* spectra, together with the unbalanced carbon content in the gaseous products (Figure 2a), may indicate the formation of carbon or graphitized materials. Altogether, these results suggest that degradative reactions such as molecular cracking and carbonization dominate in this regime, making it unlikely that selective 2‐propanol oxidation can be achieved even with the beneficial effect of water co‐feeding.

We mention that the apparent activation energies (*E_app_* ═71–74 kJ mol^−1^) during the selective regimes in dry feed were in line with the values (*E_app_* ═69 kJ mol^−1^) previously reported at ambient pressures,^[^ ^25^ ^]^ suggesting a minimal influence of the pressure gap on the catalytic functioning. These *E_app_* values (Figure 4b,c) reflect clearly the distinct regimes of acetone formation, in line with the catalyst transition. In general, the activation energy can be considered as the average energy of the transforming molecules compared to the average energy of the reactants.^[^ ^64^, ^65^ ^]^ Any contribution to the activation energy is a measure of how effectively such additional energy accelerates the dynamics of interest.^[^ ^65^ ^]^ In our case, the *E_app_* in the selective regime was found to be higher than in the non‐selective regime (22–24 kJ mol^−1^), reflecting a more pronounced potentiation of acetone production upon heating at temperatures representative of the selective regime compared to the non‐selective regime. One of the reasons for this behavior is obviously the formation of CO_2_ at expense of acetone, implying an increase of CO_2_ rather than of acetone at very high temperatures. In addition, small traces of H_2_ (See Figure S2, Supporting Information) were detected above 450 °C, which could imply a change from selective oxidation into non‐oxidative dehydrogenation paths for acetone production (see Table 1). As mentioned above, this operation regime is not beneficial due to the strong degradation of the target product.

Complementarily, we observed a variation in the ordinates of the linear Arrhenius trends during the experiments (Figure 4d). This ordinate corresponds to the sum of several terms involving intrinsic kinetic pre‐exponential factors and active sites concentration (see Note S1, Supporting Information). Accordingly, we think that these changes reflect variations in the population of active sites/species during the successive treatments. The decrease of this quantity after the phase transition and its recovery after reoxidation (Figure 4d) suggests a regeneration of the active sites, as observed structurally in the healing of the catalyst surface (Figure 3h and Figure 4a) and the recovery of the selective regime during the third heating (Figure 2a,c).

For comparison, the *E_app_* values in wet feed were 91 kJ mol^−1^ in the selective regime and 16 kJ mol^−1^ in the non‐selective regimes (Figure 6f). Interestingly, this value of 91 kJ mol^−1^ matches the previously reported activation energy for 2‐propanol oxidation in aqueous environments over cobalt spinel catalysts,^[^ ^25^ ^]^ suggesting that the same catalytic mechanisms are operative in both the liquid‐phase and the gas‐phase oxidation in a wet feed, thus establishing a strong connection between both systems. The higher *E_app_* value in the wet feed compared to the dry feed experiments indicates an enhanced potentiation of acetone formation due to the introduction of water vapor. Accordingly, the value of the Arrhenius ordinate was found to be significantly higher (Figure 6f) in wet feed than in any of the dry feed experiments, involving higher concentrations of acetone‐forming species or active sites. For the high‐temperature regimes, the *E_app_* value for acetone formation was lower in the wet feed than in the dry feed, reflecting the intense degradation of the stream at temperatures above the selective regime.

We finalize this section highlighting that, although water co‐feeding enhanced the performance in the studied system, this effect may not be universal due to several factors. For example, oxygen vacancies in certain oxides might be unresponsive to water, or their migration into the bulk may be too rapid, rendering them inaccessible to modulation by gaseous species. Therefore, further *operando* studies tailored to each system are necessary to elucidate the underlying mechanisms, for instance, by using isotopic labelling to decipher the product distribution and guide the selection of the most effective strategies. These strategies may include doping elements to tune the surface interactions, and vacancy‐engineered materials^[^ ^66^ ^]^ for improved reactivity, mobility and stability. These aspects are left for future work.

## 4 Conclusion

In summary, our study highlights that the selective oxidation of 2‐propanol is governed by a frustrated phase transition marked by the cycling of the catalyst between partially reduced and oxidized states of the spinel phase. This dynamic regime, which avoids the complete phase transition into rock‐salt, is crucial for sustaining acetone selectivity, as the intrinsic mechanisms seem to operate more effectively while the spinel structure is preserved. At 375 °C, however, fast oxygen depletion and vacancy migration generated by Mars‐van Krevelen activation destabilize the spinel phase and promote its transformation towards the rock‐salt structure, with a marked decline in acetone selectivity. The presence of water vapor in the feed mitigates this transition by rapidly refilling the vacancies and increasing the oxygen potential, thereby enhancing the acetone formation rate, extending the operational stability of the active spinel phase, and preserving the selectivity at higher temperatures. Yet, even in the wet feed, the frustrated phase transition collapses beyond 425 °C, and the catalyst transforms into rock‐salt.

Ultimately, our findings demonstrate that the frustrated transition plays a central role in controlling the reaction pathway. This insight enables the rational design of strategies to enhance the catalytic performance. However, the optimal approach must be tailored to the specific characteristics of the frustrated transition that governs each catalytic system.

## 5 Experimental Section

### OSEM Setup and Experiments

Details of our setup can be consulted elsewhere.^[^ ^30^ ^]^ We used a quartz tube reactor inside the chamber of a commercially available environmental SEM (ESEM, FEI 200 Quanta FEG) lined to a quadrupole mass spectrometer (QMS, 200 Prisma Pfeiffer). The catalyst was heated by illumination with an infrared laser (808 nm, maximum 110 W). Changes at the catalyst and in gas phase compositions are determined simultaneously, enabling the direct investigation of the influence of catalytic dynamics on performance. Our operando SEM resembles a flow reactor. Temperature and gas phase compositions were controlled during surface imaging. Pure gases (Westfalen 5.0) were dosed into the quartz tube reactor by individual mass‐flow controllers (Bronkhorst). 2‐propanol and water were dosed directly as vapors using the low‐ΔP models of the mass‐flow controllers (Bronkhorst).

Images of the catalyst surface were acquired with the large field detector of the ESEM at acceleration voltages of 7.5–10 kV at a chamber pressure of 32‐96 Pa and a pixel depth of 8 bits.

In situ *catalyst preparation*: A Co foil (99.995 %, thickness 0.2 mm, Alfa Aesar) was cut into a rectangular form (8 × 5 mm^2^, 71.2 mg) and a K‐type thermocouple was spot‐welded onto its surface before mounting the sample inside the quartz tube reactor. Before the experiments, the OSEM chamber and gas feeding system were evacuated to remove any residual gas and moisture inside the system. Then, the Co foil was oxidized inside the OSEM at 615 °C for 1 h under a continuous flow of Ar (3mL_N_min^−1^) and O_2_ (1mL_N_min^−1^) and a chamber pressure of 32 Pa. Subsequently, the sample was cooled down to room temperature.

*2‐propanol oxidation in dry feed*: The reactant feed composed of Ar (3mL_N_min^−1^), O_2_ (3mL_N_min^−1^), and 2‐propanol (3mL_N_min^−1^) was introduced to the OSEM reactor at room temperature, resulting in a chamber pressure of 62 Pa. The gas feed was kept overnight until steady signals were observed in the QMS. The catalyst was then thermally treated under constant reactant feed in four consecutive runs.

In the first run (heating‐1), starting from room temperature the sample was heated up to 100 °C and then in steps of 50 °C, up to 200 °C, following a further heat‐up in steps of 25 °C up to the final temperature of 500 °C. In the second run (cooling), the same temperature profile was executed as a cool‐down of the catalyst, starting from an initial temperature of 500 °C down to room temperature. In the third run (heating‐2), the heat‐up procedure from the first run was repeated in order to characterize the reversibility of the observed processes. Afterwards, the catalyst was reoxidized in a mixture of Ar (3mL_N_min^−1^) and O_2_ (1mL_N_min^−1^) and a chamber pressure of 32 Pa at 615 °C for 1 h and then allowed to cool‐down to room temperature. The reactant feed gas mixture was reintroduced in the OSEM reactor. In the fourth final run (heating‐3), the catalyst was heated‐up in the reactant feed following the same protocol used in the previous runs.

*2‐propanol oxidation in wet feed*: This experiment was performed in the OSEM reactor using a gas composition of Ar (1mL_N_ min^−1^), O_2_ (3 mL_N_min^−1^), water (2mL_N_ min^−1^), and 2‐propanol (3mL_N_ min^−1^) at a pressure of 62 Pa to investigate the effect of water vapor in the reaction. The heating protocol was the same as used in the first run (heating‐1).

### NAP‐XPS Experiments

The soft branch of the beamline UE56/2 PGM‐1 (Elliptical Undulator) at Bessy II was used for the near‐ambient‐pressure X‐ray photoelectron spectroscopy (NAP‐XPS) measurements. The soft X‐ray beamline was operated with a monochromator equipped with a 800 l mm^−1^ grating and an exit slit of 10 µm, resulting in nominal band widths of 60 and 100 meV for O‐K and Co‐L edges, respectively. A differentially pumped Specs Phoibos 150 NAP hemispherical analyzer, coupled to a 2D delay line detector, was used to detect photoelectrons with a pass energy of 10 eV.

Potential offsets from the monochromator were corrected for by the valence band (inflection point), which was recorded at every photon energy and, additionally at hν = 150 eV, where any offset from the monochromator was negligible. For Co*2p* spectra, any additional residual shifts (e.g., from the lens system) were removed by cross‐correlation of the spectra. All shift‐correction measures were additionally cross‐checked with the C*1s* binding energies of the C‐C component (at 284.8 eV).

A chamber pressure of 50 Pa was used at the end station. For oxidation steps, the chamber was filled with pure oxygen, while, for reactions, 1:1 mixtures of 2‐propanol vapor (dosed via a low‐Δp mass flow controller, Bronkhorst) and O_2_ were used (flows of 2 mL min^−1^ each), which were additionally mixed with a small flow of argon (0.2 mL min^−1^). The metallic Co sample was mounted in a sapphire sample holder and oxidized in situ at 600 °C for 1 h. Before the reaction in the presence of 2‐propanol, the sample was allowed to cool down to room temperature, and subsequently the reaction gas mixture was admitted in the chamber. The heating was achieved by illumination from the back of the sample holder using a near‐infrared laser (808 nm, 60 W). The temperature was measured with a K‐type thermocouple (mounted on the spectrometer‐facing side of the pellet) and kept constant using a Eurotherm PID controller.

### Post‐Catalytic TEM Characterization

After the experiment in the OSEM, a thin cross‐section (lamella) of the spent catalyst was prepared with an FEI Helios NanoLab G3 focused ion beam (FIB)/scanning electron miscrosope (SEM) system using a Ga ion beam. While the cross‐section was cut, images of the sample were taken using the SEM. The prepared lamella was further investigated using a double aberration‐corrected JEOL JEM‐ARM 200CF transmission electron microscope (TEM) operated at 200 kV.

### Theoretical Analysis

All DFT calculations employed VASP with PAW–PBE+U (Dudarev U_eff_ ═ 3.32 eV on Co *3d*, J ═0), spin polarization, ENCUT═ 520 eV (PREC═Accurate), ISMEAR═ −5, SIGMA═ 0.05 eV, LASPH, and LMAXMIX═ 4; bulk relaxations used EDIFF═ 10−5, IBRION═ 2, ISIF═ 3, KSPACING═0.25 A^−1^, LREAL═Auto. CoO was initialized in AFM‐II with an even number of {111} planes; Co_3_O_4_ in the normal‐spinel cation order with magnetic Co^2+^ at 8*a* and (nominally) nonmagnetic Co^3+^ at 16*d*. Migration barriers were computed via CI‐NEB with IBRION═ 3, SPRING═ −5, POTIM═ 0.1, EDIFFG═ −0.02 eV A^−1^, 5 images (CoO) and 5–9 (Co_3_O_4_); transition states were checked by single‐ended dimer and finite‐difference frequencies. The optimized hosts reproduced the AFM‐II distortion in CoO and the normal‐spinel topology in Co_3_O_4_. Vacancy formation energies were converged against plane‐wave cutoff, *k*‐point density, and supercell size.

## Conflict of Interest

The authors declare no conflict of interest.

## Supporting information



Supporting Information

Supplemental Video 1

Supplemental Video 2

Supplemental Video 3

Supplemental Video 4

Supplemental Video 5

Supplemental Video 6

Supplemental Video 7

Supplemental Video 8

Supplemental Video 9

## Data Availability

The data that support the findings of this study are openly available in AC/CATLAB archive at https://ac.archive.fhi.mpg.de/D63409, reference number 63409.
