# Supplementary material for: Stabilizing Frustrated Phase Transitions in Selective Oxidation Reactions
Source: Adv Mater. 2025 Nov 24;38(8):e15292. doi: 10.1002/adma.202515292 (PMC12879286; doi:10.1002/adma.202515292)
Supplement: Supplementary file 1 — Supporting Information [file ADMA-38-e15292-s001.docx]

Supporting Information

Stabilizing Frustrated Phase Transitions in Selective Oxidation Reactions

Luis Sandoval-Diaz^*^, Thomas Götsch, Daniel Cruz, Maurits Vuijk, Juan M. Lombardi, Markus Pietsch, Kassiogé Dembélé, Adnan Hammud, Karsten Reuter, Christoph Scheurer, Axel Knop-Gericke, Thomas Lunkenbein^*^

Part 1. Experimental

**supplementary note 1.**

Eq. 1 represents the Arrhenius equation of the apparent kinetic constant.

$k\left( T \right)=Ae^{-\frac{E_{app}}{RT}}$ (1)

${r(T)}_{acetone}=k\left( T \right)\left[ 2-propanol \right]^{m}{[O_{2}]}^{n}{[\theta]}^{o}$ (2)

In Eq. 2, *θ* represents the abundance of active sites on the catalyst, and *m*, *n* and *o* represent the reaction orders of 2-propanol, O_2_, and the active sites, respectively.

In our flow reactor, the rate of acetone formation is strictly proportional to *Δacetone* at each temperature, i.e., the difference between the QMS signal of acetone at any time on stream, and the value of this signal at the beginning of the experiment, when no acetone was formed. Hence,

${r(T)}_{acetone}={Cal}_{acetone}\Delta acetone(T)$ (3)

Here, *Cal_acetone_* is the experimentally determined calibration sensitivity factor of acetone in the QMS.

A combination of Eq. 1-3 gives the following logarithmic equation:

$\ln\left( \Delta acetone \right)=lnA+mln\left( [2-propanol] \right)+nln\left( [O_{2}] \right)+oln\left( \theta\right)-ln{(Cal}_{acetone})-\frac{E_{app}}{RT}$ (4)

From which the apparent activation energy of acetone formation, *E_app_*, can be estimated. The intercept of this function on the vertical axis would be:

$Arrhenius Ordinate=lnA+mln\left( [2-propanol] \right)+nln\left( [O_{2}] \right)+oln\left( \theta\right)-ln{(Cal}_{acetone})$ (5)


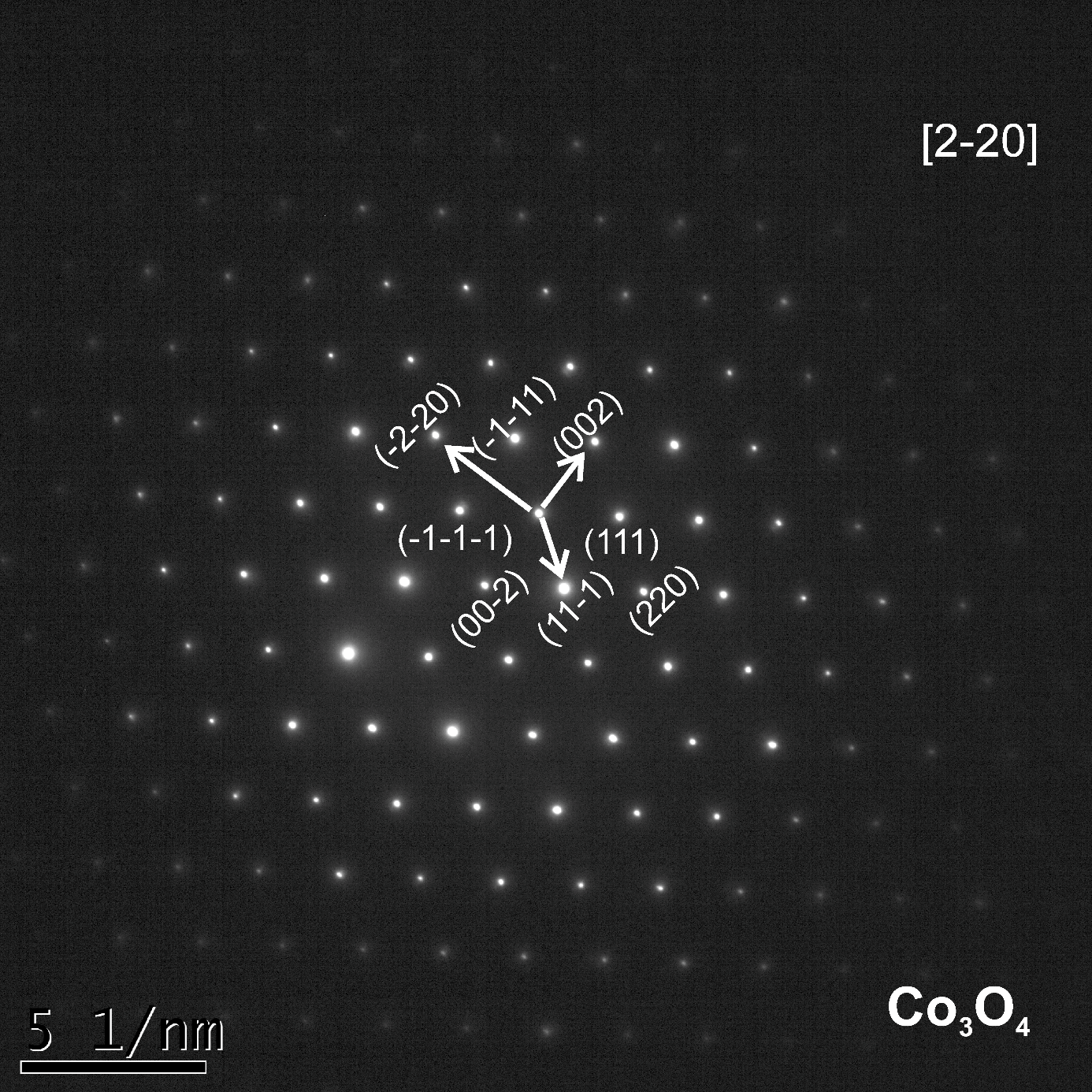


**Fig. S1. Selected area electron diffraction (SAED) pattern of the pristine cobalt oxide catalyst prepared by in situ oxidation of a metallic cobalt foil at 615°C in the OSEM reactor.**

**Fig. S2. Time series of the QMS reaction traces (ionic currents) during the gas phase oxidation of 2-propanol in dry feed measured in the OSEM reactor.** From bottom to top: Reaction temperature, and QMS traces normalized to Ar for 2-propanol, oxygen, acetone, propylene, water, carbon dioxide, carbon monoxide, and hydrogen. Time axis break between 35h and 38h represent the reoxidation step after the second heating treatment. Data acquired in 3Nccmin^-1^ 2-propanol: 3Nccmin^-1^ O_2_: 3Nccmin^-1^ Ar.

**Supplementary note 2.**

Fig. S2 summarizes the catalytic data collected in the OSEM reactor during 2-propanol oxidation in dry feed, i.e., without any addition of water vapor in the reaction stream, over the oxidized Co foil. The figure depicts the time series of the experiment including the temperature of the reactor, the traces detected by quadrupole mass spectrometry (QMS) of the educts (2-propanol, i-POH) and O_2_), and the products expected from the different reactions pathways including oxidative dehydrogenation (acetone), dehydration (propylene), combustion (CO_2_, CO), and dehydrogenation (H_2_), respectively. The temperature was stepwise increased to 500 °C (TOS=15-23h). Afterwards, the temperature was decreased stepwise (TOS=23-28h) in a similar manner. Subsequently, the system was heated again (TOS=29-35h) to 500 °C. At TOS= 35h-38h, the catalyst was reoxidized in 25%O_2_ atmosphere at 615 °C, and heated for a third time (TOS= 38h-47h) in the reaction feed.


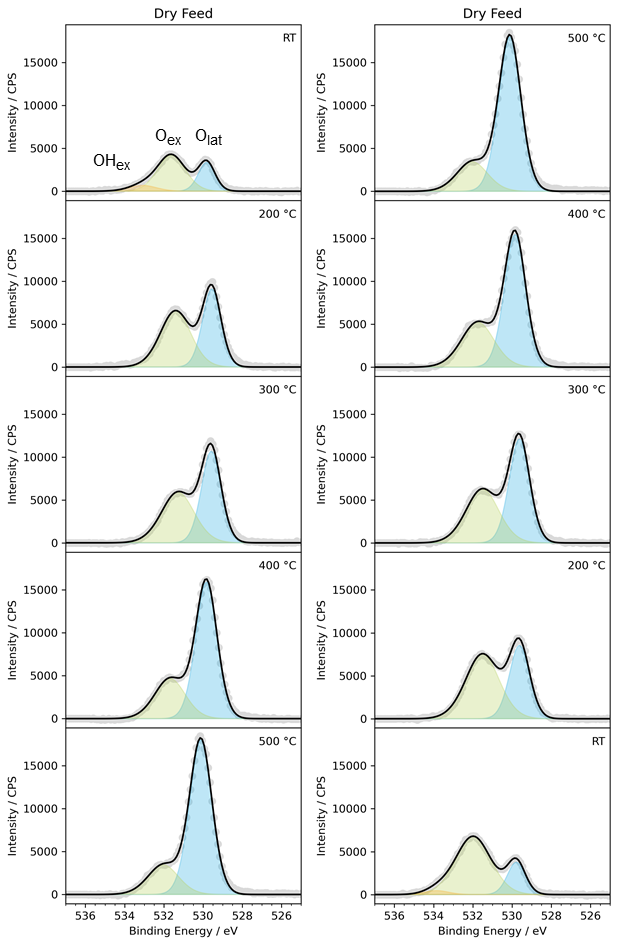


**Fig. S3. NAP-XPS spectra of O1s as a function of temperature during 2-propanol oxidation in a dry feed.** Data acquired in 3Nccmin^-1^ 2-propanol: 3Nccmin^-1^ O_2_: 3Nccmin^-1^ Ar.


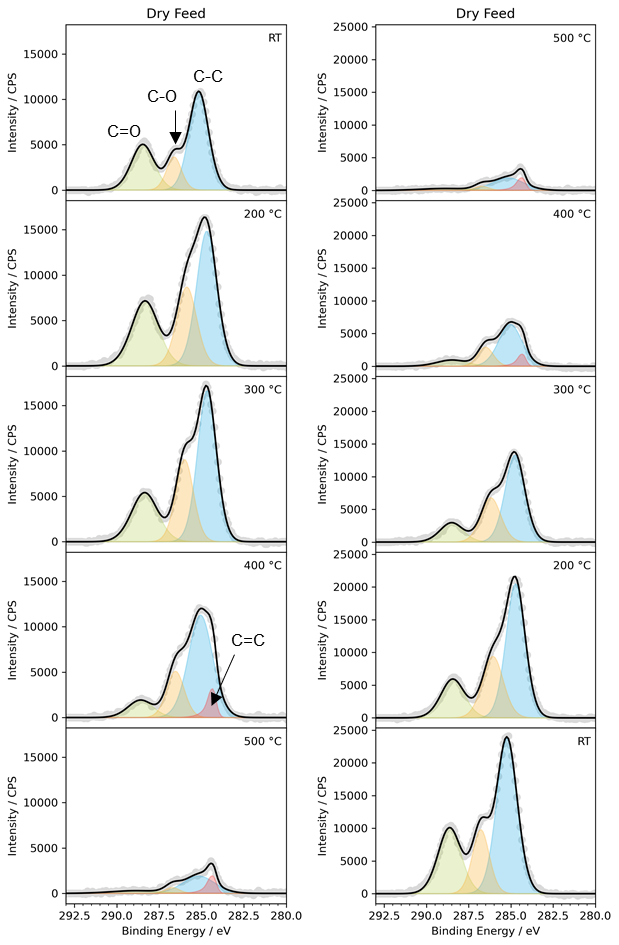


**Fig. S4. NAP-XPS spectra of C1s as a function of temperature during 2-propanol oxidation in a dry feed.** Data acquired in 3Nccmin^-1^ 2-propanol: 3Nccmin^-1^ O_2_: 3Nccmin^-1^ Ar.

**Fig. S5. Time series of the QMS reaction traces (ionic currents) during the gas phase oxidation of 2-propanol in wet feed measured in the OSEM reactor.** From bottom to top: Reaction temperature, and QMS traces normalized to Ar for 2-propanol, oxygen, acetone, propylene, water, carbon dioxide, carbon monoxide, and hydrogen. Data acquired at 62 Pa in 3Nccmin^-1^ 2-propanol: 2Nccmin^-1^ H_2_O: 3Nccmin^-1^ O_2_: 1Nccmin^-1^ Ar.


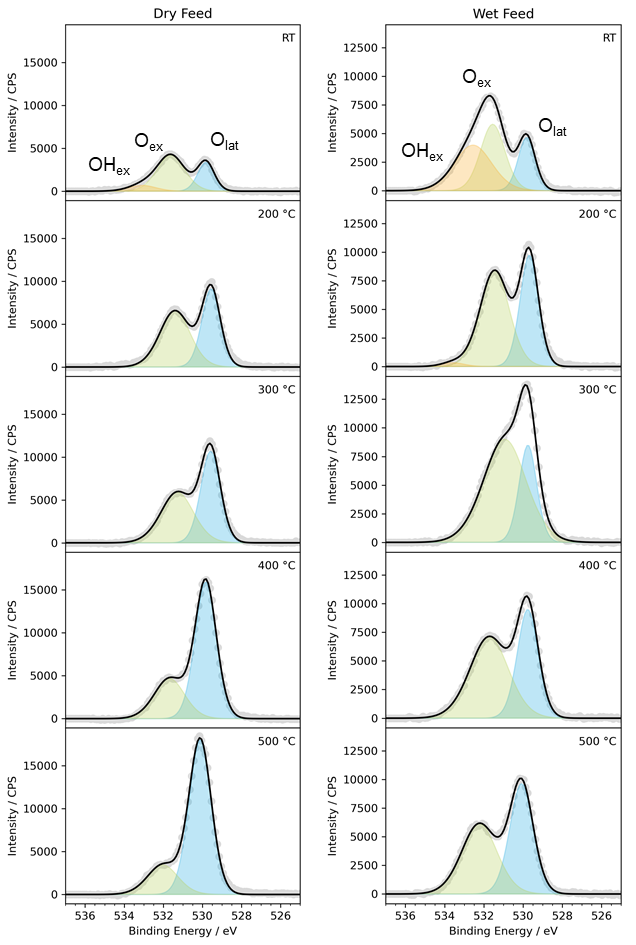


**Fig. S6. A comparison of NAP-XPS spectra of O1s as a function of temperature during 2-propanol oxidation in wet and dry feeds.** Wet feed data acquired in 3Nccmin^-1^ 2-propanol: 2Nccmin^-1^ H_2_O: 3Nccmin^-1^ O_2_: 1Nccmin^-1^ Ar.


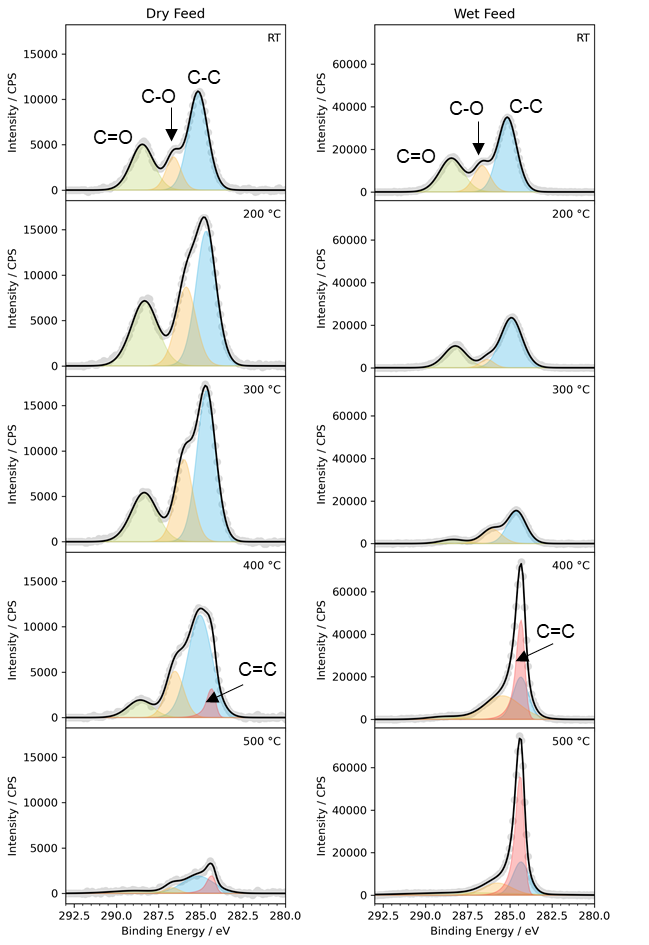


**Fig. S7. A comparison of NAP-XPS spectra of C1s as a function of temperature during 2-propanol oxidation in wet and dry feeds.** Wet feed data acquired in 3Nccmin^-1^ 2-propanol: 2Nccmin^-1^ H_2_O: 3Nccmin^-1^ O_2_: 1Nccmin^-1^ Ar.


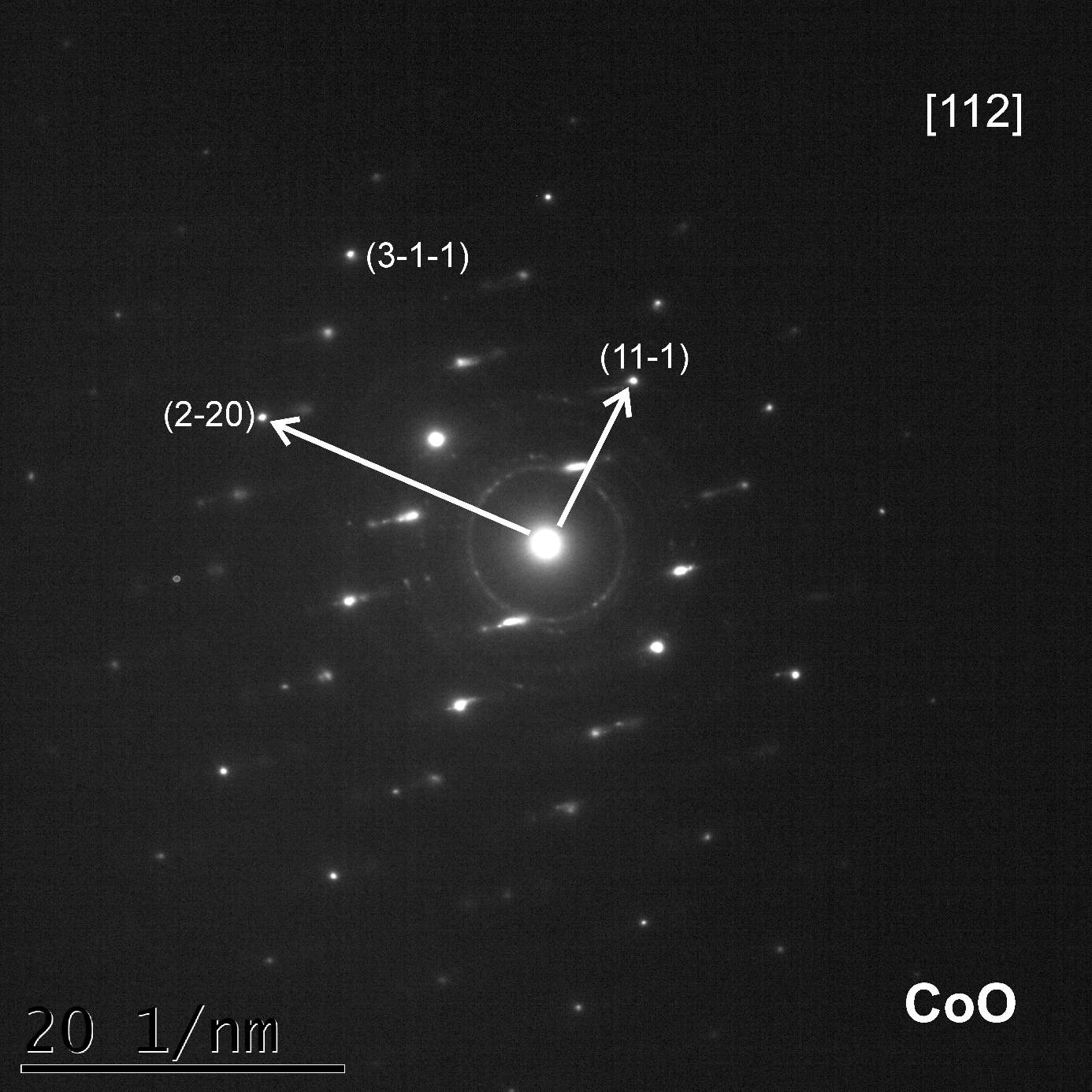


**Fig. S8. Selected area electron diffraction (SAED) pattern of the post catalytic sample obtained after gas-phase 2-propanol oxidation in dry feed.**


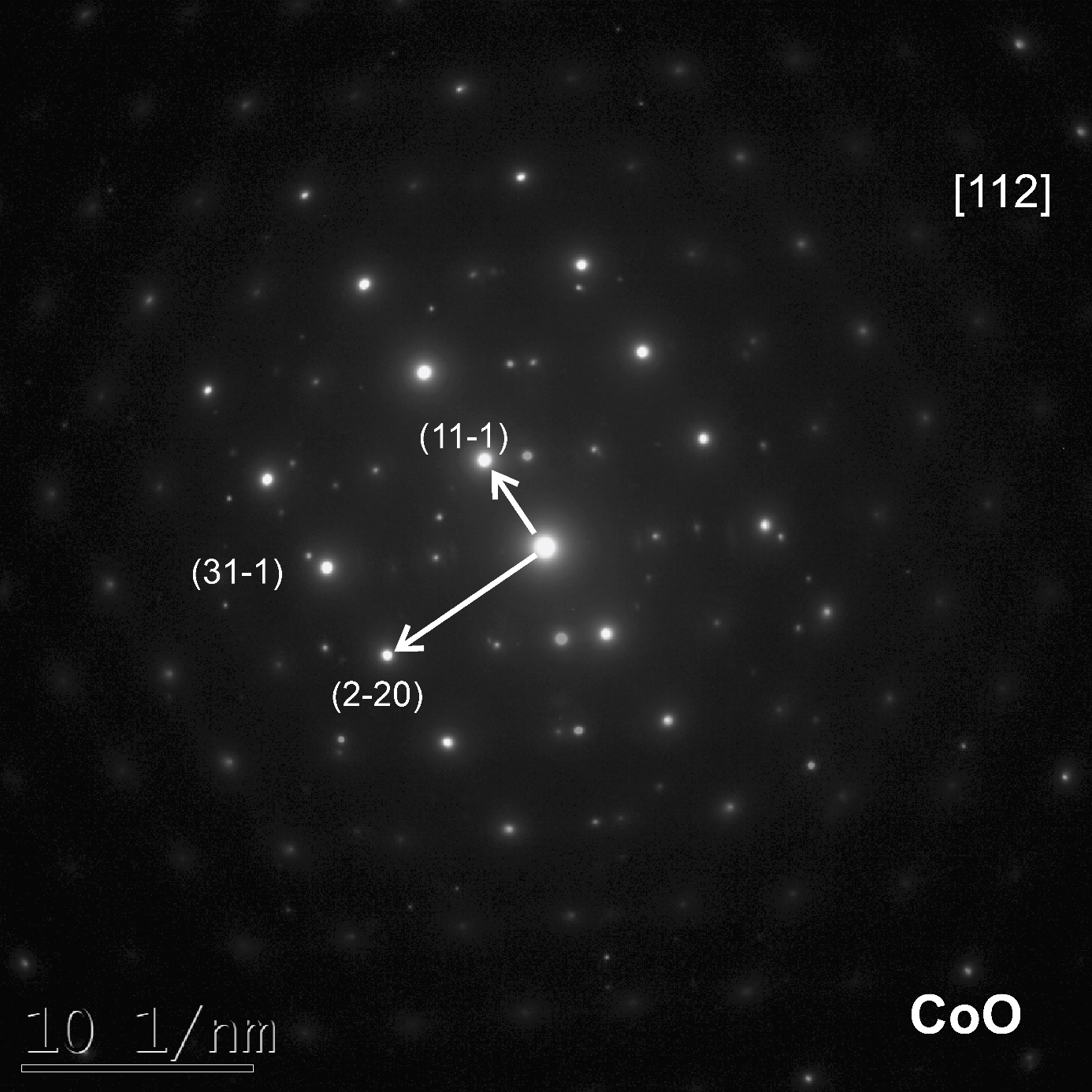


**Fig. S9. Selected area electron diffraction (SAED) pattern of the post catalytic sample obtained after gas-phase 2-propanol oxidation in wet feed.**

Part 2: Ab Initio Thermodynamics and Vacancy Transport in Bulk CoO and Co_3_O_4_

# Crystal Structures and Model Supercells for CoO and Co_3_O_4_

**CoO (rock salt, AFM-II).** Bulk CoO adopts the NaCl (rock-salt) lattice (space group *Fm*3¯*m*) at high temperature. Below the Neel transition (*T*_N_ ≈ 290 K) it orders in the canonical *type-II antiferromagnetic* structure consisting of ferromagnetic {111} planes stacked + − + − along ⟨111⟩ [[1].](#_bookmark17) The magnetic ordering is accompanied by a small symmetry-lowering distortion; high-resolution diffraction shows a predominantly monoclinic (*C*2*/m*) low-temperature struc- ture with a weak tetragonal component [[2].](#_bookmark18) For supercell modeling we therefore (i) construct AFM-II cells with an *even* number of stacked {111} planes to satisfy periodic parity; (ii) apply opposite initial moments on alternating planes; and (iii) relax ions and lattice vectors, which reproducibly recover the AFM-II ground state and the expected distortion [pattern.[1,](#_bookmark17) [2]](#_bookmark18)

**Co**_3_**O**_4_ **(normal spinel).** Co_3_O_4_ crystallizes in the normal spinel structure (space group *Fd*¯3*m*) with Co^2+^ on tetrahedral 8*a* sites (high spin, *S*=3*/*2) and Co^3+^ on octahedral 16*d* sites (low spin, *S*=0); oxygen occupies 32*e* with the usual internal parameter *u* ≈ 0*.*26 [[3,](#_bookmark19) [4].](#_bookmark20) The bulk is an antiferromagnet with *T*_N_ ∼ 30–40 K; magnetism arises from the Co^2+^ sublattice while octahedral Co^3+^ remains nonmagnetic [[5].](#_bookmark21) In our spin-polarized calculations we initialize moments on tetrahedral Co^2+^ only, preserving the normal-spinel cation order. Supercells are built by isotropic replication of the *Fd*3¯*m* conventional cell to accommodate point defects and migration paths without spurious self-interaction.

These structure and magnetism choices match neutron-diffraction and first-principles literature and underpin the convergence and NEB analyses reported in the [SI.[1–5]](#_bookmark21)

# Thermodynamic Framework and Vacancy Formation: Humidity- Controlled *µ*_O_⇒∆*G*_vac_

We reference the oxygen chemical potential to the standard free energy of water formation, avoiding any explicit DFT treatment of O_2_. Consider

$\frac{1}{2}O_{2}+H_{2}\rightleftharpoons H_{2}O$, $\Delta G_{rxn}^{0}\left( T \right)= \mu_{H_{2}O}^{0}\left( T \right)-\mu_{H_{2}}^{0}\left( T \right)-{\frac{1}{2}\mu}_{O_{2}}^{0}(T)$ (6)

Eliminating the standard chemical potential of O_2_ gives the absolute oxygen potentials, i.e. $\mu_{O}$expressed per O atom, in both wet and dry reservoirs:

$\mu_{O}^{wet}\left( T,p \right)=\mu_{H_{2}O}^{0}\left( T \right)-\mu_{H_{2}}^{0}\left( T \right)-{\Delta G}_{rxn}^{0}\left( T \right)+k_{B}T ln\frac{pH_{2}O}{p^{0}}{-k}_{B}T ln\frac{pH_{2}}{p^{0}}$ (7)

$\mu_{O}^{dry}\left( T,p \right)=\mu_{H_{2}O}^{0}\left( T \right)-\mu_{H_{2}}^{0}\left( T \right)-{\Delta G}_{rxn}^{0}\left( T \right)+\frac{1}{2}k_{B}T ln\frac{pO_{2}}{p^{0}}$ (8)


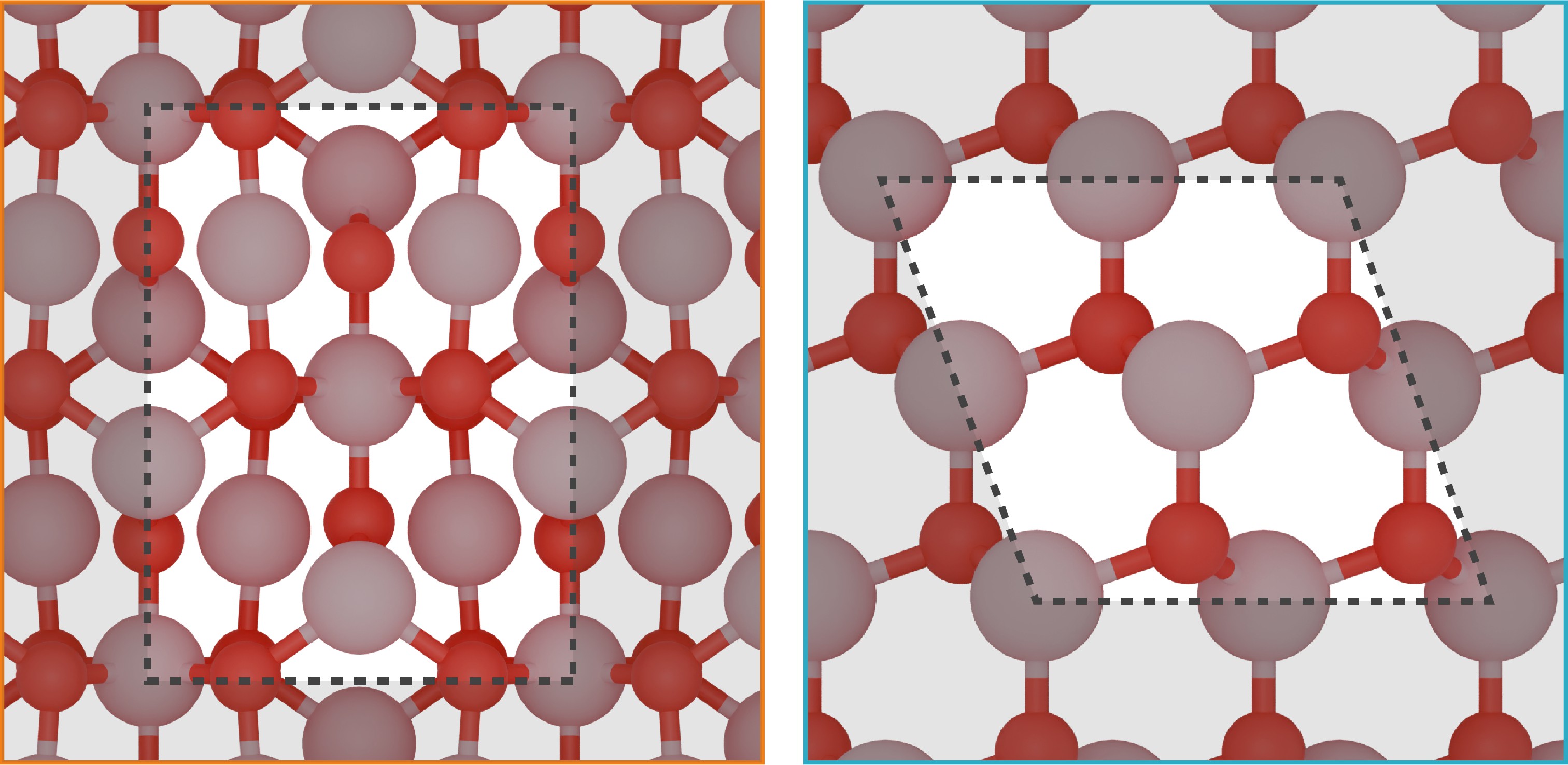


**Figure S10: Structural models used in this work for CoO (rock-salt, left) with AFM-II {222} stacking and Co_3_O_4_ (normal spinel, right). Co (pink), O (red).**

At fixed *T* , the **wet–dry** difference cancels all standard-state terms:

$\Delta\mu_{O}^{wet-dry}\left( T \right)=k_{B}T ln\frac{pH_{2}O}{pH_{2}}-\frac{1}{2}k_{B}T ln\frac{pO_{2}}{p^{0}}$  (9)

**Numerical bounds for the experimental feeds**

Using the OSEM selective-window conditions (*p*_tot_ ≈ 62 Pa), with *p*^wet^ (H_2_O) ≈ 0*.*30 *p*_tot_ and *p* (H_2_) below detection (bounded at 10^−4^–10^−3^ Pa),

2

we obtain

∆*µ*^wet−dry^(375 ^◦^C) ∈ [0*.*549*,* 0*.*678] eV*,*

O

∆*µ*^wet−dry^(425 ^◦^C) ∈ [0*.*592*,* 0*.*731] eV*,*

O

∆*µ*^wet−dry^(500 ^◦^C) ∈ [0*.*654*,* 0*.*807] eV*.*

O

- 1. **Vacancy formation free energy under humidity**

For a neutral bulk oxygen vacancy,

∆*G*_vac_(*T, p*) = *E*_def_ − *E*_bulk_ + *µ*_O_(*T, p*)*.* (10)

From the DFT totals,

∆*G*^CoO^ = (−9*.*132) + *µ*_O_*,* (11)

vac

∆*G*^Co^3O4 = (−8*.*907) + *µ*_O_*,* (12)

vac

so the *phase-intrinsic* offset is

(13)

∆*G*

Co O

3 4

CoO

vac

— ∆*G* = +0*.*226 eV*.*

vac

Because *µ*_O_ enters linearly, the wet–dry shift in formation energy is *identical* for both phases:

(14)

∆*G*wet − ∆*G*dry = ∆*µ*wet−dry*.*

vac

vac

O

Thus, at fixed *T* the equilibrium vacancy ratio is

$\frac{c_{v}^{wet}}{c_{v}^{dry}}=e^{-\frac{{\Delta\mu}_{O}^{wet-dry}}{k_{B}T}}$ (15)

which evaluates to 10^5^–10^6^ suppression at 375 ^◦^C for the bounds above.

# Vacancy Migration Kinetics

**Hop topology.** Oxygen vacancies migrate on the anion sublattice by nearest-neighbour hops. In rock-salt CoO the active path connects two fcc O sites across an edge of the Co_6_ octahedral cage (a ⟨110⟩-like hop), yielding a comparatively open saddle. In normal-spinel Co_3_O_4_ the hop links 32*e* oxygen sites through a tighter bottleneck framed by edge-sharing Co(tet)/Co(oct) polyhedra, which sterically and electronically stiffens the transition state.

**CI-NEB barriers.** Climbing-image NEB gives activation energies

*E_a_*(CoO) = 1*.*62 eV*, E_a_*(Co_3_O_4_) = 2*.*93 eV*,* (16)

with transverse forces converged to
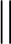
 **F
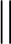
** ≤ 0*.*02 eV ˚A^-1^ using 5 images for CoO and 5–9 images for Co_3_O_4_ (Fig. [2).](#_bookmark14) Refining image density or *k*-meshes within the convergence window changes *E_a_* by only a few×10 meV; barriers are insensitive to the AFM-II plane parity in CoO and to modest variations of *U*_eff_ .

⊥

**Diffusivity contrast and temperature scaling.** Approximating vacancy diffusivity as

*D_v_* ≃ *a*^2^*ν* exp(−*E_a_/k_B_T* ) with similar attempt frequencies *ν*, the ratio is governed by ∆*E_a_* =

2*.*93 − 1*.*62 = 1*.*31 eV:

$\frac{D_{v}^{{Co}_{3}O_{4}}}{D_{v}^{CoO}}\cong e^{-\frac{{\Delta E}_{a}^{spinel-rock salt}}{k_{B}T}}=\left\{ \begin{aligned} {6.5x10}^{-11} at 375{}^{o}{C, k_{B}T=0.0559eV} \\ {3.5x10}^{-10} at 425{}^{o}{C, k_{B}T=0.0559eV} \\ {2.9x10}^{-9} at 500{}^{o}{C, k_{B}T=0.0559eV} \end{aligned} \right.$ (17)

Thus, *before* thermodynamic suppression is considered, vacancy motion in the spinel is slower by 9–11 orders of magnitude across the experimental window. Since the vacancy flux satisfies *J_v_* ∝ *c_v_D_v_*, humidity (which lowers *c_v_*) and the intrinsic kinetic penalty (small *D_v_*) act multiplicatively to choke long-range reduction in Co_3_O_4_.

# Mechanistic Summary

Humidity serves as a tunable oxygen buffer: each decade increase in *R* ≡ *p*_H_2O*/p*_H_2 raises the oxygen potential by *k*_B_*T* ln 10 [[](#_bookmark2)[Eq.](#_bookmark4) (4)], which increases ∆*G*_vac_ by the same amount [[Eq. (9)]](#_bookmark8) and suppresses the vacancy population *c*_v_ ∝exp[−∆*G*_vac_*/*(*k*_B_*T* )] by the factor *R*_dry_*/R*_wet_ [[Eq. (10)].](#_bookmark9) Because Co_3_O_4_ carries a phase-intrinsic +0*.*226 eV higher vacancy formation cost than CoO [[Eq. (8)]](#_bookmark7) and a much larger migration barrier (∼ 1*.*31 eV higher; [Fig.](#_bookmark14) 2), the net vacancy flux *J*_v_ ∝ *D*_v_*c*_v_ is doubly reduced in the spinel under wet feeds. The combined thermodynamic and kinetic penalties delay the Co_3_O_4_ → CoO reduction and extend the selective acetone window under the OSEM conditions considered.


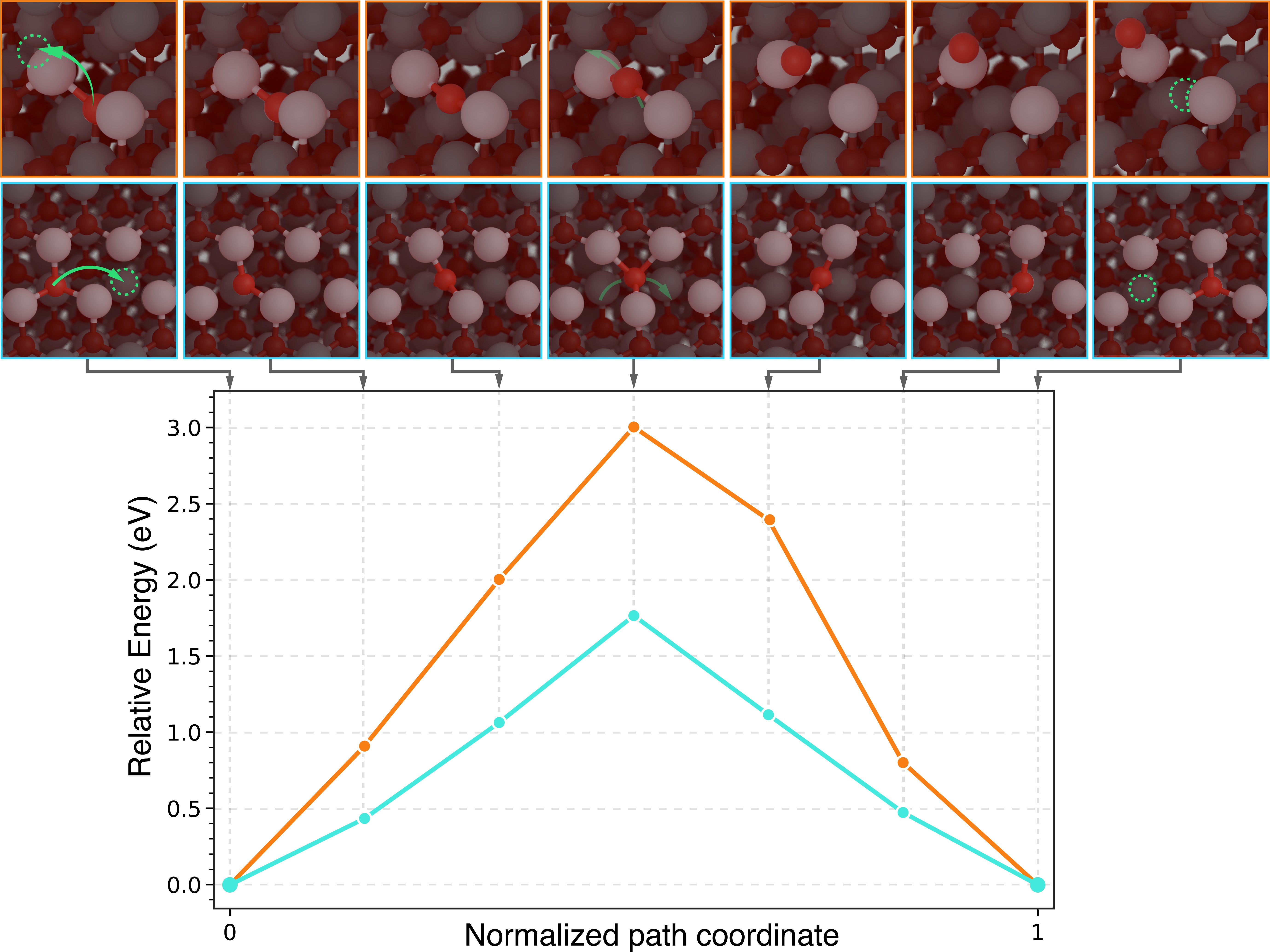


**Fig. S11.** **CI-NEB results.** Top: images along the minimum-energy path in CoO (cyan) and Co_3_O_4_ (orange). Bottom: relative energies vs. normalized path coordinate showing *E_a_* =

1*.*62 eV (CoO) and 2*.*93 eV (Co_3_O_4_).

Because ∆*G*_vac_ = (*E*_def_ − *E*_bulk_) + *µ*_O_ [[Eq.](#_bookmark6) (5)], the same increment applies to vacancy forma-

tion [[Eq.](#_bookmark8) (9)], yielding *c*^wet^*/c*^dry^ = exp[−∆*µ_O_/*(*k_B_T* )] and the sensitivity *∂* ln *c/∂* ln *R*=−1

[[Eq. (10)].](#_bookmark9) Under the experimental bounds, this corresponds to a 10^5^–10^6^ suppression of *c*_v_ at 375 ^◦^C and several decades at higher *T* .

Independently of gas composition, Co_3_O_4_ carries a phase-intrinsic +0*.*226 eV higher vacancy formation cost than CoO [[Eq. (8)]](#_bookmark7) and a much larger migration barrier (approximately 1.31 eV higher), so that the ratio of the diffusivities is 10^-9^-10^-11^ smaller in the spinel compared to the rock salt [[Eq. (11)].](#_bookmark11) Hence the vacancy flux *J*_v_ ∝ *D*_v_*c*_v_ is multiplicatively reduced in spinel under wet feeds: humidity lowers *c*_v_ while lattice topology yields small *D*_v_. This coupling explains the delayed Co_3_O_4_ →CoO reduction and the extended selective acetone window under the OSEM conditions.

**Design levers (forward-looking).** (i) Gas-phase control of *R* deterministically tunes *µ*_O_;

(ii) temperature ramps modulate the balance between thermodynamic suppression (*c*_v_) and kinetic activation (*D*_v_); (iii) lattice/defect engineering (e.g., cation substitution, controlled non- stoichiometry) that lowers the spinel migration barrier can partially offset humidity-induced flux suppression without sacrificing selectivity; (iv) microstructure optimization (short diffusion lengths, oriented grains) reduces the effective *L*^2^*/D*_v_ penalty, particularly impactful for Co_3_O_4_.

# Computational Details (Reproducibility)

All DFT calculations employed VASP with PAW–PBE+U (Dudarev *U*_eff_ = 3*.*32 eV on Co 3*d*, *J* =0), spin polarization, ENCUT= 520 eV (PREC=Accurate), ISMEAR= −5, SIGMA= 0*.*05 eV, LASPH, and LMAXMIX= 4; bulk relaxations used EDIFF= 10^−5^, IBRION= 2, ISIF= 3, KSPACING=

0*.*25 ˚A−1, LREAL=Auto. CoO was initialized in AFM-II with an *even* number of {111} planes;

Co_3_O_4_ in the normal-spinel cation order with magnetic Co^2+^ at 8*a* and (nominally) nonmagnetic Co^3+^ at 16*d*. Migration barriers were computed via CI-NEB with IBRION= 3, SPRING= −5, POTIM= 0.1, EDIFFG= -0.02 eV A^-1^, 5 images (CoO) and 5-9 images (Co_3_O_4_). Transition states were checked by single-ended dimer and finite-difference frequencies. Optimized hosts reproduce the AFM-II distortion in CoO and the normal-spinel topology in Co_3_O_4_.

# Numerical Convergence: Cutoff, *k*-Density, and Supercell Size

Vacancy formation energies were converged against plane-wave cutoff, *k*-point density, and supercell size. Increasing ENCUT beyond 520 eV changes ∆*G*_vac_ by only a few*meV* (Fig. [3);](#_bookmark22) a reciprocal-space density corresponding to KSPACING= 0*.*25 A˚−1 yields similar residuals (Fig. [4).](#_bookmark23)

Supercell tests (respecting the CoO AFM-II even-plane constraint) show a clear plateau once several tens of formula units are included, indicating negligible defect–image interactions (Fig. [5).](#_bookmark24) We therefore adopt ENCUT= 520 eV, KSPACING= 0*.*25 A˚−1, and parity-consistent supercells for

production.

**Fig. S12: Convergence of the *relative* vacancy formation energy with plane-wave cutoff (ENCUT).** Variations beyond 520 eV remain within a few*meV* , establishing 520 eV as a safe production value.

**Fig. S13. Convergence with *k*-point density.** A density corresponding to KSPACING = 0*.*25 ˚A−1 (or finer) yields formation-energy variations at the level of a few *meV* , adequate for defect thermodynamics and barrier comparisons.

**Fig. S14. Convergence with supercell size (number of formula units).** AFM-II imposes an even count of {111} planes; within that constraint, vacancy formation energies plateau once the cell hosts several tens of formula units, indicating negligible finite-size interactions.

# References

1. W. L. Roth. Magnetic structures of MnO, FeO, CoO, and NiO. *Physical Review*, 110: 1333–1341, 1958. doi: 10.1103/PhysRev.110.1333.
2. W. Jauch, M. Reehuis, H. J. Bleif, F. Kubanek, and P. Pattison. Crystallographic symmetry and magnetic structure of CoO. *Physical Review B*, 64:052102, 2001. doi: 10.1103/PhysRevB.64.052102.
3. Jia Chen, Xifan Wu, and Annabella Selloni. Electronic structure and bonding properties of cobalt oxide in the spinel structure. *Physical Review B*, 83:245204, 2011. doi: 10.1103/ PhysRevB.83.245204.
4. Thomas D. Sparks and David R. Clarke. High-temperature structure of Co 3O 4: Under- standing spinel stoichiometry. *Journal of the American Ceramic Society*, 102(7):3620–3630, 2019. doi: 10.1111/jace.16242. Preprint/PDF widely available.
5. Swati R. Gawali, Ashish C. Gandhi, Shrikrushna S. Gaikwad, Jayashree Pant, Ting-Shan Chan, Chia-Liang Cheng, Yuan-Ron Ma, Sheng Yun Wu, et al. Role of cobalt cations in short-range antiferromagnetic Co 3O 4 nanoparticles: a thermal treatment approach to affecting phonon and magnetic properties. *Scientific Reports*, 8:249, 2018. doi: 10.1038/ s41598-017-18563-9.
